# Epidemiology and Economic Burden of Chikungunya: A Systematic Literature Review

**DOI:** 10.3390/tropicalmed8060301

**Published:** 2023-05-31

**Authors:** Lourrany Borges Costa, Francisca Kalline de Almeida Barreto, Marina Carvalho Arruda Barreto, Thyago Henrique Pereira dos Santos, Maria de Margarette Oliveira de Andrade, Luís Arthur Brasil Gadelha Farias, André Ricardo Ribas de Freitas, Miguel Julian Martinez, Luciano Pamplona de Góes Cavalcanti

**Affiliations:** 1 Programa de Pós-Graduação em Saúde Coletiva, Universidade Federal do Ceara (UFC), Ceara 60020-181, Brazil; lourranybc@unifor.br (L.B.C.);; 2 Faculdade de Medicina, Universidade de Fortaleza (UNIFOR), Ceara 60811-905, Brazil; 3 Hospital São Jose de Doenças Infecciosas, Ceara 60455-610, Brazil; 4 Faculdade de Medicina, Centro Universitário Christus (UNICHRISTUS), Ceara 60192-345, Brazil; 5 São Leopoldo Mandic, Campinas 13045-755, Brazil; 6 Microbiology Department, Hospital Clínic-Universitat de Barcelona, 08036 Barcelona, Spain; 7 Barcelona Institute for Global Health (ISGlobal), Hospital Clínic-Universitat de Barcelona, 08036 Barcelona, Spain; 8 Centro de Investigación Biomédica en Red de Enfermedades Infecciosas (CIBERINFEC), Instituto de Salud Carlos III, 28220 Madrid, Spain

**Keywords:** chikungunya, outbreak, epidemiology, incidence, economic impact

## Abstract

Chikungunya (CHIK) is a re-emerging viral infection endemic in tropical and subtropical areas. While the typical clinical presentation is an acute febrile syndrome, long-term articular complications and even death can occur. This review characterizes the global epidemiological and economic burden of chikungunya. The search included studies published from 2007 to 2022 in MEDLINE, Embase, LILACS, and SciELO for a thorough evaluation of the literature. Rayyan software was used for data analysis, and data were summarized descriptively and reported following the Preferred Reporting Items for Systematic Reviews and Meta-Analyses (PRISMA) guidelines. Seventy-six publications were included. Chikungunya is widely distributed in the tropics, including Africa, Asia, South America, and Oceania/the Pacific Islands, and co-circulates with other simultaneous arboviruses such as DENV, ZIKV, and YFV. Chikungunya infection can lead to chronic articular manifestations with a significant impact on the quality of life in the long term. In addition, it generates absenteeism and economic and social losses and can cause fatal infections in vulnerable populations, mainly in high-risk patients with co-morbidities and at the extremes of age. Reported costs associated with CHIKV diseases are substantial and vary by region, age group, and public/private delivery of healthcare services. The chikungunya disease burden includes chronicity, severe infections, increased hospitalization risks, and associated mortality. The disease can impact the economy in several spheres, significantly affecting the health system and national economies. Understanding and measuring the full impact of this re-emerging disease is essential.

## 1. Introduction

Chikungunya is a re-emerging acute viral infection characterized by fever; intense arthralgia, which can progress to systemic complications and death; and common musculoskeletal manifestations. It can progress to long-term chronic conditions with sequelae [1,2,3,4]. The Chikungunya virus (CHIKV) belongs to the *Alphavirus* genus within the *Togaviridae* family. Transmission to humans usually occurs through the bite of an infected mosquito, mainly *Aedes aegypti* and *Aedes albopictus* [5,6,7]. Genetic analyses of strains have identified three main distinct lineages of CHIKV: the West African lineage, the East/Central/South African (ECSA) lineage, and the Asian lineage [5].

From the first reported cases in 1952 in Tanzania to 2021, CHIKV infection has been detected in more than 100 countries, and millions of cases have been reported worldwide [5,6,8,9]. Since the resurgence of the virus in 2004, more than 70 epidemics and sporadic outbreaks of CHIKV have been reported in different parts of the world, mainly in Africa, Asia, and regions of the Pacific Ocean. In the Western Hemisphere, the first infection was reported in 2013 on the island of Saint Martin, with rapid diffusion to other Caribbean islands, reaching 45 countries in the Americas by 2015 [4,7,10]. Outbreaks or locally acquired cases in non-dengue-endemic areas have also been reported, and the virus represents a public health concern for *Ae. Albopictus*-colonized areas such as many European countries [11,12].

Recent studies suggest that the impact of the chikungunya disease is exceptionally high, leading to absenteeism and economic costs, mainly due to its extraordinary epidemic potential and associated joint pain, which can be severe and disabling [13,14,15]. Moreover, CHIKV represents a serious public health threat to non-endemic areas where competent *Aedes* vectors are established. However, despite the relevance and the proven re-emergence of CHIK, few studies have assessed the economic impact of this disease. 

This systematic review aimed to assess current evidence regarding the quality of life impact, economic burden, and mortality associated with CHIKV infections. The epidemiology, pertaining to outbreaks and the spread of the disease across the globe, is also addressed.

## 2. Materials and Methods

### 2.1. Study Design, Search Strategy, and Article Selection

The review was conducted according to the Cochrane Handbook for Systematic Reviews and the PRISMA guidelines [16,17]. The search included all original studies published in English, Spanish, or Portuguese, from 2007 to 2022, in the following databases: MEDLINE^®^ (via PubMed), Embase, LILACS, and SciELO. We used the following search terms, their equivalents in Portuguese and Spanish, and their combinations: “Chikungunya”, “Cross-Sectional Studies”, “Cohort Studies”, “Morbidity”, “Mortality”, “Disability-Adjusted Life Years”, “Seroepidemiologic Studies”, “Cost of Illness”, “Cost Allocation”, “Health Care Costs”, “Drug Costs”, “Direct Service Costs”, “Hospital Costs”, “Cost Efficiency Analysis”, “Cost-Benefit Analysis”, and “Cost Analysis”. This systematic review was registered on PROSPERO (International Prospective Register of Systematic Reviews) with registration number CRD42022350256, and the search strategy is detailed in the Supplementary Materials (Table S1).

### 2.2. Eligibility Criteria and Study Selection

Studies were included in the review if they reported the following primary outcomes: (1) epidemiological data on chikungunya, such as prevalence, incidence, and seroprevalence, or information on the severity of chikungunya (morbidity, mortality, and hospitalization); (2) disease cost and economic burden of chikungunya for patients and health services and/or social factors, such as direct medical costs, direct non-medical costs, and indirect social costs. We considered studies describing CHIKV infection in all age groups. 

We searched for randomized and non-randomized controlled study designs, non-randomized controlled trials, cohort studies, case-control studies, cross-sectional studies, outbreak reports, genomic studies, systematic literature reviews if a meta-analysis was included, and cost of illness or disease burden studies. Case reports and reviews were used as sources of references only. Publications that did not clearly describe the methods and sources for data collection and analysis, in vitro studies, studies providing non-human data, studies focusing exclusively on imported cases of chikungunya, cost-effectiveness models, clinical trials that did not report baseline and/or analyze placebo/control outcomes, and news and opinion articles were not considered for the systematic review. A free reference manager software, Mendeley (https://www.mendeley.com (accessed on 21 December 2022)), and the website Rayyan (http://rayyan.qcri.org (accessed on 26 July 2022)) was used to sort the articles, accounting for duplicates, the organization of references, practicality, and time optimization. The full text of the selected studies in the screening was recovered.

### 2.3. Data Extraction and Synthesis

Two review authors independently read titles and abstracts and excluded articles with irrelevant titles or abstracts from further analysis (having identified the clear exclusion of features). The articles selected by each reviewer were compared, and disagreements were resolved through consensus or consultation with a third investigator. After reading the full articles, the reviewers made a final selection based on exclusion criteria. At this stage, disagreements were resolved by consensus or consultation with a third reviewer. Finally, the selected review articles’ references were examined to find additional potentially eligible studies not identified in the database searches. The author names, language, study setting, year, study population, research design, objectives, and main results of the selected articles were organized in tables. The data were summarized based on the region and population’s key characteristics and findings.

### 2.4. Quality Assessment

The quality of individual articles was assessed based on the application of standardized checklists for each selected article [18]. Two independent reviewers evaluated the study’s risk of bias and quality using: (1) the Newcastle–Ottawa Scale for observational studies, including cohort and case-control studies [19]; (2) the Joanna Briggs Institute (JBI) Critical Appraisal Checklist for analytical cross-sectional studies [20]; (3) the Consolidated Health Economic Evaluation Reporting Standards (CHEERS) statement for health economic evaluation and cost studies [21]; and (4) A Measurement Tool to Assess Systematic Reviews 2 (AMSTAR-2) for systematic reviews and meta-analysis studies [22]. 

## 3. Results 

**Figure 1:**
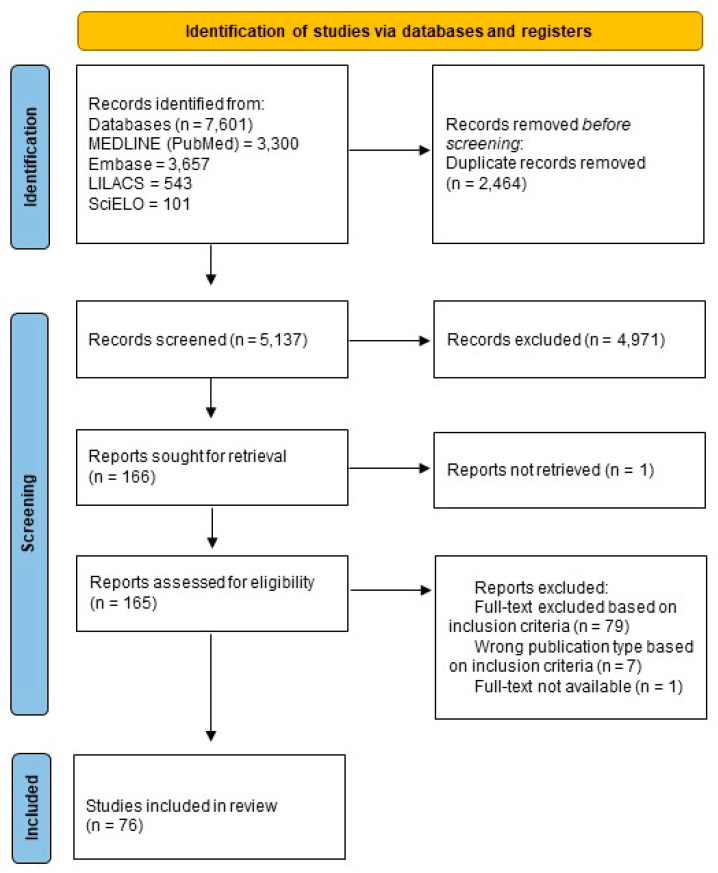
PRISMA flow diagram showing a schematic illustration of database searches and identification, screening, and eligibility of included studies.

The systematic review began in July 2022 in the MEDLINE (via Pubmed), Embase, LILACS, and SciELO databases and found 7601 references. After the removal of duplicates, 5137 references remained. Of these, 166 were selected initially by title and abstract. After analyzing full texts, 76 articles were selected according to established inclusion criteria (Figure 1). 

**Figure 2:**
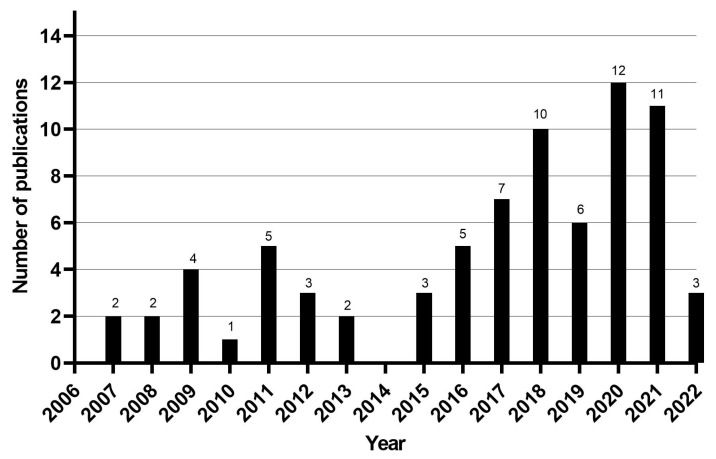
Number of articles per year of publication.

Out of the 76 articles included in the review, 66 (87%) were original research articles, 6 (7.8%) were review articles, and 4 (5.2%) were short communications. Most articles were written in English (n = 72; 93.5%), while only two (2.6%) were written in Portuguese and three (3.9%) in Spanish. Among the selected articles, 64 (83.1%) were epidemiological studies (51 cross-sectional studies, five prospective cohort studies, two retrospective cohort studies, one cross-sectional followed by a prospective clinical cohort study, and five systematic reviews with meta-analysis), and 12 were economic evaluation studies. Most studies were published between 2016 and 2021 (Figure 2) and performed in Asia, Africa, and South America; more specifically, in India, Brazil, La Réunion Island, and Colombia. The detailed extraction of articles is presented in a cell format, and the consensus results of the risk of bias assessment for epidemiology and cost studies are displayed in tables (Supplementary Materials Tables S2 and S3, respectively).

### 3.1. Regional Epidemiology

#### 3.1.1. Africa

Twenty articles reporting CHIKV seroprevalence in Africa were included. The most frequently mentioned countries were La Réunion, Tanzania, Kenya, and Nigeria.

The first cases of chikungunya were reported on the Réunion Islands, in the African region, with seven articles included in the review overall. The surveillance system estimated 244,000 cases of CHIKV infection between March 2005 and April 2006, with an overall attack rate of 35%. Nearly every case reported by sentinel physicians was accompanied by fever (96.3%) and joint pain (96.6%). In addition, 203 death certificates indicating CHIKV infection were obtained, with a median age of 79 [23]. After 2005, the virus spread further, affecting nearby countries. Studies in African regions showed a current prevalence between 30 and 70% [24,25,26,27,28,29,30,31,32,33,34,35].

In a study in the Mayotte archipelago, the seroprevalence was 37.2% [25]. Nearby, on Comore Island, the seropositivity was 68% (139 of 204) [26]. The same authors also investigated another island in Kenya; among 288 serum samples tested, 75% were positive for antibodies against CHIKV [27]. In another study in the same country, the seroprevalence was 34% [28].

In the Republic of Congo, 178 out of 517 blood donors (34.4%) tested positive for IgG anti-CHIKV [29]. A seroprevalence of 43.6% was found in Ethiopia [30]. In turn, studies in Mozambique showed a seroprevalence of 28.6% (112/392) for anti-CHIKV IgG [31]. An extensive meta-analysis, including cross-sectional studies conducted in Nigeria, identified a pooled anti-CHIKV IgM and IgG seroprevalence of 26.7% and 29.3%, respectively (*n* = 1347) [32]. In another *Alphavirus* and *Flavivirus* seroprevalence survey conducted in 2022 in Nigeria, 290 (41.3%) of the 701 samples tested were seropositive for CHIKV [33]. 

In Tanzania, different cross-sectional studies revealed an increased seroprevalence over time. The seroprevalence raised from 7.9% [34] in 2012 to 14% in 2018 [35] and 28% in 2021, with even greater differences between some districts, reaching 46% in the case of Buhigwe [24]. 

Significant differences in seroprevalence were observed according to gender. Females were more likely to be affected, with odds ranging from 1.45 to 1.87 (95% CI 1.07–2.38, *p* < 0.0001) [25,26,30,32]. Interestingly, low educational attainment was also associated with a higher risk of CHIKV exposure, with odds ranging from 1.68 to 2.74 (95% CI 1.06–3.95, *p* < 0.0001) [25,28,32]. 

The higher the age, the higher the exposure, with an odds ratio of 2.15 (*p* < 0.001, 95% CI: 1.33–3.45) among adults [33]. Moreover, in the study of Endale et al. (2020), the seroprevalence was 53.5% among the 36–55 age group (OR = 5.37, 95% CI, 1.44–20.03) [30]. However, two studies found higher exposure in younger patients, such as the 1- to 4-year-old group [34,35].

In a study with an emphasis on rheumatic symptoms, the most frequently reported symptoms were polyarthralgia (99%), muscular pain (93%), backache (86%), and abrupt-onset fever (85%) [25]. 

Since long-term symptoms have been described, Schilte et al. (2013) followed 190 patients for 36 months and found that arthralgia was intermittent in 25–40% of the patients. Just 31% fully recovered from acute symptoms. Arthralgia (usually symmetrical) caused stiffness in 75.5% of patients. Local swelling, cutaneous symptoms, myalgia, and osteoligamentous pain also occurred. Soumahoro et al. (2009) and Gérardin et al. (2011) paired positive and negative chikungunya cases to identify factors that worsened the disease or caused sequelae. The CHIKV seropositive subjects under 30 reported faster recovery than the older subjects, and these patients reported more fatigue, light cerebral disorders, and sensorineural impairment than CHIKV-seronegative peers. Attention, memory, mood, sleep, and depression were the light cerebral disorders most linked to CHIKV infection [36,37,38].

Some cases of CHIKV infection can be more severe and require hospitalization. In the study of Sergon et al. (2007), 79% of the cases were hospitalized or stayed at home in bed for a mean of 6 days (range 1–30 days), and 52% missed work or school for a mean of 7 days (range 1–40 days). Importantly, patients with severe CHIKV who required hospitalization or life support were those who had comorbidities such as hypertension, diabetes mellitus, cardiovascular diseases, and neurological disorders. Many of them needed intensive care. Age was one of the major risk factors associated with higher-severity cases [39].

Several attempts have been made to assess pain and the extent to which it impacted patients’ lives, both in acute and chronic phases. In the study by Andrade et al. (2010), the mean pain intensity on the visual analog scale (VAS) was 5.8 ± 2.1, and its duration was 89 ± 2 days. According to the DN4 questionnaire, many patients reported chronic and neuropathic pain features. These patients experienced more interference in daily life and provided low scores on the affective and social levels. The worst score was found in patients aged 30 to 59 and females [37].

#### 3.1.2. Asia

The highest number of articles were published in Asia, with the vast majority in India (12 articles), followed by Pakistan, Thailand, and Bangladesh. Among these countries, we found different seroprevalences, with 5.93% in Malaysia [40]; 18–29% in Bangladesh [41,42]; 18–36% in India [43,44,45]; 34% in Myanmar; 71% in Thailand (1295 reagents from the 1806 samples tested) [46]; and 80% in Bangladesh [41]. Studies that compared prevalence over time observed increases. For example, the study by Aubry et al. (2020) in Fiji found that the prevalence increased from 0.9% (95% CI 0.2–2.6%) in 2015 to 12.8% (95% CI 9.4–17%) in 2017 [47]. Asymptomatic infections were only reported in the study of Dutta et al. (2019), accounting for 17.86% of the cases [48].

Males were more likely to be CHIK-seropositive in Malaysia [40]. This was also found by Hossain et al. [42] in Bangladesh; Barr [49] and Badar et al. [50] in Pakistan; Dutta et al. [48], Joshi et al. [51], and Chopra et al. [52] in India. In contrast, the studies of Chattopadhyay et al. [44] and Ramachandran et al. [53] in India found higher rates in females, than in Thailand [46,54].

In several studies, exposure to CHIKV was found to increase with time, and the average age of the cases was around 30 to 40 years old [40,43,51,54,55,56].

More detailed descriptions of the symptoms were found. On the Asian continent, fever was the most prevalent symptom, appearing in between 84 and 100% of reported cases [44,46,48,50,53]. Arthralgia was the second most frequently reported symptom [42,53], first affecting the spine and the small and large joints of the extremities with no upper/lower limb predilection [57]. The pain was usually bilateral. Rashes were generalized, erythematous, and maculopapular [41,43]. Headache was among the most cited symptoms, followed by joint swelling and abdominal pain [43,48,50]. Fatigue was only reported by Chopra et al. [57], whereas tachycardia was reported by Barr et al. [49], along with laboratory alterations such as high levels of aspartate aminotransferase and lymphopenia. Gastrointestinal disturbances and symptoms of the central nervous system were also reported.

Only three studies mentioned the hospitalization of patients. The hospitalization rate stayed between 5.7% to 41%, with a hospital stay duration ranging from 2 to 15 days [42,44,48]. Furthermore, only two studies followed up with patients to determine recovery and chronic pain over time: Dutta et al. [48] perceived that 42% complained about sequelae of infection. In the study by Chopra et al. [57], 16% of patients continued to suffer beyond four months.

Some studies investigated patients’ recovery and chronic pain. In the study of Mathew et al. [52], the most typical diagnosis was chronic post-viral polyarthralgia (57%), with the most typical pain site being the knee (83.3%), followed by the ankle, low back, shoulder, and wrist. Many of these patients experienced wrist, ankle, and hand swelling. A subgroup of these patients was subjected to musculoskeletal ultrasonography, demonstrating tenosynovitis and bursitis.

Regarding factors that could worsen the progression of the disease and the quality of life of patients affected by chikungunya, Ramachandran et al. [53] reported that age, duration of fever, multiple joint afflictions, duration of joint pain, and duration of joint swelling were significantly and negatively associated with Health-Related Quality of Life (HRQoL) scores for various domains. Age, employment type, and severe arthralgia significantly affected the QoL in the study of Hossain et al. [42]. The average score was highest in the environmental health domain, followed by the psychological domain, the social relationship domain, and the physical domain, which were all substantially affected, indicating a significant impact on the quality of life during acute-phase CHIKV infection.

#### 3.1.3. Caribbean and Central America

Ten articles included in this review were studies from the following Caribbean areas: Saint Martin, Martinique, Guadeloupe, Curaçao, Puerto Rico, Nicaragua, Aruba, and the U.S. Virgin Islands. 

Saint Martin Island reported the first CHIKV autochthonous transmission in America in 2013. From 6 December 2013 to 5 December 2014, Sint Maarten, the Dutch part of the island, reported 658 chikungunya cases; 238 (61%) were women. Fever (71%) and arthralgia (69%) were the most common symptoms. The attack rate was 1.76%, considered to be underestimated, as some cases may have been misdiagnosed as dengue [58].

Since then, several locations have reported cases. The first CHIKF cases were observed in Martinique and Guadeloupe in December 2013. From January 2014 to January 2015, 36% of Martinique’s population—representing approximately 145,000 cases—was infected [59]. Seroepidemiological surveys in blood donors revealed that the final seroprevalence was 48.1% in Guadeloupe and 41.9% in Martinique [60]. The first U.S. Virgin Islands CHIKV infection occurred in June 2014. A study estimated a 31% (95% CI: 26–36%) infection rate [61]. Passive surveillance in Puerto Rico found 28,327 cases in 2014; 6472 were screened for CHIKV, and 4399 (68%) were positive. In the household cluster studies, 70 (28%) of the 250 participants had recently been infected with CHIKV. Detecting the virus in blood or tissue samples revealed 31 fatal cases [62].

Nicaragua first reported chikungunya in September 2014. The anti-CHIKV antibody seroprevalence was 33% and the clinical attack rate was 26.5% in 11,280 blood samples from 39 locations (37 municipalities and 2 districts of Managua, the capital) in October 2015. Of these, 19.1% had subclinical infections [63]. Another study in Managua, from 2014 to 2016, examined 2327 children aged 2 to 14 for CHIKV. After 95 cases in 2014–2015, a larger wave occurred in 2015–2016 (444 cases). The cohort included 81.6 CHIKV cases per 1000 person-years (95% CI: 75.0–88.8). The study found that CHIKV prevalence increased with age [64].

Studies have investigated the severity of CHIKV infection in several Caribbean countries. From December 2013 to January 2015, researchers studied CHIKV-related severe cases and mortality in four public hospitals in Martinique and Guadeloupe. Of 1836 hospitalized cases, 64.8% were adults (15+ years old), with a mean age of 41 and a sex ratio of 0.9. Ten mother-to-child transmissions were detected in Guadeloupe and five in Martinique. The overall incidence rate of hospitalization was 23.4/10,000 inhabitants. The attack rate of hospitalization was 60/10,000 CHIKV clinical cases. The incidence rates were highest in the elderly (>75 years old) and infants (< 1 year old) (296 and 80/10,000, respectively). A total of 74 people died because of CHIKV infection. Fifty-one percent of hospitalized cases had an underlying health problem [65]. Crosby et al. observed 65 CHIKV patients in ICUs at university hospitals in Martinique and Guadeloupe in 2014 [66]. Forty-one percent were admitted for comorbidity aggravation, and 83% had pre-existing conditions. Admission to ICUs and mortality rates were 26 and 27%, respectively. Twenty-eight (18%) had CHIKV-related symptoms, including encephalitis, Guillain–Barré syndrome, and severe sepsis [66].

Couzigou et al. [59] followed 509 Martinique residents from January 2014 to January 2015 to evaluate CHIKF aggravation factors. The female-to-male ratio was 1.98, with an average age of 43.2 years. Twenty-three percent of the patients had unusual or severe acute chikungunya infection risk factors. Three months after acute chikungunya infection, 200 subjects (39.3%) showed signs of chronic infection. Remission patients were younger than symptomatic patients (*p* < 0.0001). More than half (55.8%) struggled to resume daily activities. They had a much lower QoL (median: 71; range: 0–100) than remission patients (median: 90; range: 40–100) (*p* < 0.0001). Another study found that 52.10% (95% CI 44.5–59.7) of 167 CHIKV-infected Martinique hospital patients had chronic chikungunya arthritis (CCA) after 12 months. In the univariate analysis; age; female sex; and some clinical signs at disease onset, such as headache, vertigo, vomiting, and dyspnea, increased the probability of CCA [67]. In Aruba, 55% of 489 patients tested from October 2014 to April 2015 were positive for CHIKV, and 44% had chronic arthralgia [68]. In Curaçao, 30–50% of residents were infected by CHIKV in June–July 2014, only 43% of 248 patients recovered >2.5 years after disease onset, and 22% had severe chronic arthralgia. Highly affected patients had more persistent rheumatic and non-rheumatic/psychological symptoms and lower physical and mental QoL than mildly affected patients [1].

#### 3.1.4. South America

CHIKV quickly spread to South America from the Caribbean. Sixteen South American articles were reviewed. A study carried out to determine the frequency of Zika (ZIKV), chikungunya (CHIKV), and dengue (DENV) virus co-infection during the epidemiologic surveillance of the ZIKV epidemic in Colombia analyzed 23,871 samples from suspected Zika cases. The frequency of CHIKV was only 1.07% [69]. From 2015 to 2016, 45% of 319 Ecuadorian blood samples tested positive for CHIKV, and seroprevalence averaged 27% (95% CI: 8.7–51.6%) [70]. From June to October 2017, 2697 people from 22 French Guiana municipalities participated in a multiplexed serological survey; 20.3% (17.7–23.1) were CHIKV-positive [71].

In Brazil, the first autochthonous chikungunya cases were recorded in 2014, almost simultaneously in the semi-arid region of the Brazilian Northeast, Feira de Santana in Bahia, and the Amazon Forest Region Oiapoque in Amapá. 

Fifty-seven percent of 385 Feira de Santana residents tested positive for CHIKV antibodies, and 68.1% had chronic chikungunya. In Riachão do Jacuípe, 10 km from Feira de Santana, 45.7% of 446 participants had CHIKV antibodies and 75.0% developed the chronic form of the disease [72]. A comparable study was undertaken in Chapada, in April 2016,Of the 120 tested individuals, 18.3% presented anti-CHIKV IgG, 5.0% IgM, and 40.7% CHIKV symptoms [73]. In a 2016–2017 Feira de Santana study, 22.1% of the 1981 people tested (95% CI 16.7–28.6) had CHIKV [74]. Among 451 people from two indigenous populations of the São Francisco Valley—the Fulni-ô and Truká—and an urbanized control community from Juazeiro, a large city in Bahia, a CHIKV IgG prevalence of 49.9% was found [75]. Salvador, Bahia’s capital, was also the subject of a cross-sectional seroprevalence study. Of 2651 eligible study site residents, 1776 (67.0%) participated from November 2016 to February 2017; 11.8% (95% CI 9.8%–13.7%) had CHIKV IgG [76].

The northeast of Brazil was the most affected region of the country. In 2017, Ceará had the highest incidence rate, with 139,729 reported and 105,312 confirmed cases (1174.9 per 100,000 inhabitants). Fifty CHIKV-related deaths occurred in 2016, one hundred and ninety-four in 2017, and one in 2018 [77]. A primary care clinic in Fortaleza, Ceará, examined the sociodemographic and clinical parameters of 110 chikungunya patients in 2018. Sixty percent of the patients were female; their average pain score was 6.81 (±2.49), and 60.91% used medications. In addition, pain, age, time since diagnosis, and educational level affected QoL [78]. In 2018, CHIKV, DENV, and ZIKV seroprevalences were estimated in Juazeiro do Norte, a large city in southern Ceará. Four hundred and four volunteers were analyzed; 25.0% of them were CHIKV seropositive [79]. The CHIKV seroprevalence was 18.0% (95% CI 14.8–21.2) in 2120 Rio de Janeiro residents tested for arbovirus antibodies between July and October 2018 [80]. 

Vidal et al. found a national case-fatality rate of 0.13% for chikungunya in Brazil in 2016, with an incidence rate of 114.70/100,000 and a mortality rate of 0.15/100,000. In 2017, these values were 87.59, 0.12/100,000, and 0.14%, respectively. In 2016, Brazil lost 77,422.61 DALYs or 0.3757/1000 people, and in 2017, 59,307.59, or 0.2856/1000 people [81]. The official national surveillance systems (SINAN and SIM) reported 552,023 chikungunya cases between 2014 and 2017, with 403 deaths and a lethality rate (LR) of 0.7/1000 cases. Frutuoso et al. found 552,023 SINAN chikungunya cases in 2016–2017. By linking SINAN and SIM data, 3135 CHIKF-related deaths were found. CHIKV was listed on 764 death certificates, and 17.6% died from CHIKF. Most deaths occurred in the acute (38.1%) and post-acute (29.6%) stages. The corrected lethality rate (CLR) was 6.8 times higher than SINAN alone (0.8/1000). CLR and death risk were higher for residents in the Northeast region (6.2); men (7.4); those under one year (8.6), 65–79 years (20.7), and 80 years of age (75.4); those with a low level of education (none: 16.8; 1–3 years: 33.7); and those who were White (14.6) or Black (11.1) [3].

#### 3.1.5. Worldwide Reviews

Three systematic reviews with meta-analyses were included. Together, these articles investigated the worldwide seroprevalence of CHIK and its chronic symptoms, mainly chronic arthralgia.

Li et al. included 44 articles with 51,599 participants from 29 countries and regions. The CHIKV seroprevalence was 25% (95% CI: 22–29). South-East Asia had the highest seroprevalence (42%, 95% CI: 17–67), whereas the Eastern Mediterranean region had the lowest (2%, 95%: 0–5). Infection rates were highest in Cameroon, Comoros, Haiti, Thailand, and Indonesia. Compared to 2000–2009, global seroprevalence dropped in 2010–2019 [82].

Badawi et al. performed a systematic review and meta-analysis to determine the frequency of chronic comorbidities in CHIKV patients and their potential effects on infection severity and complications. Eleven studies, including 2773 patients, were selected from 111 articles. Hypertension was the most common comorbidity in CHIKV infection (31.3%), followed by diabetes (20.5%), cardiac disorders (14.8%), and asthma (7.9%). One study reported obesity prevalence. Diabetes was present in 22.9% vs. 20.5% of severe CHIKV cases (*p* < 0.05). Infected patients with diabetes (including types I and II) but not hypertension or cardiac illness had an OR of 1.2 (95% CI: 1.05–1.48; *p* = 0.0135) for severe CHIKV outcomes compared to those without diabetes [83]. 

Rodríguez-Morales et al. conducted a systematic review to identify studies assessing the proportion of patients progressing to chronic inflammatory rheumatism (CIR) following CHIKV infection. Eighteen studies were included, reporting data on 5702 patients. The pooled prevalence of CHIK-CIR was 40.22% (95% CI 31.11–49.34; τ2 = 0.0838). The prevalence of chikungunya chronic arthritis was 13.6% (95% CI 9.31–18.00; τ2 = 0.0060) [84]. 

### 3.2. Costs Studies

Few studies considered the effective costs of chikungunya. Data on economic impacts collected from the articles analyzed are shown in Table 1.

Regarding the loss in productivity caused by CHIKV, a study carried out in India estimated 7.4 million lost days, considering only acute episodes of the disease. Such a situation would have an estimated cost of between INR 214.4 and 391 million (corresponding to USD 2.57–4.69 million) [85]. In the same location, some patients reported absence from work for up to 35 days, representing an estimated loss of USD 75 in income [86]. In the Reunion Islands, the loss in productivity generated a loss of EUR 17.4 million (corresponding to USD 18.79 million) [87]. In Bangladesh, approximately 70% of patients missed more than 7 days of work, while 29.6% of them missed more than 10 days, considering only the acute phase of the disease [42].

A loss in productivity was also reported in the Americas. In Colombia, the average cost of lost productivity reached USD 81.3 (USD 72.2–203.2) per adult patient [88]. In Mexico, in 2015, the cost of disability was greater than USD 180,000 and, in 2014, greater than USD 130,000 [89]. In the US Virgin Islands, after 1–2 months of illness onset, CHIKV-related absenteeism cost approximately USD 713–825 per person, USD 275–318 after six months, and USD 148–172 one year later. Absenteeism after 1 year of illness cost USD 1.76 million [90].

Considering disability-adjusted life years (DALYs), a study carried out in India by Krishnamoorthy et al. estimated 25,588 days lost, with acute episodes contributing to 7909 DALYs (30.9%) and persistent disabling arthralgia representing 69.1% [85]. In Colombia, in the period 2013–2016, CHIIKV caused 71.3% of DALYs (350,531.62). When the chikungunya epidemic peaked in 2015, Colombia lost 290,033.7 DALYs [91]. Another author pointed out that the chronic phase was responsible for 96% of lost DALYs nationwide, representing an estimated 39 to 43 days lost per 100,000 inhabitants [13]—a scenario similar to that of Brazil in the same decade [92].

Regarding the direct costs of the disease, a study in India estimated a value between USD 30 and 141, highlighting the cost of diagnosis [86]. In the Reunion Islands, for only 1 year, the costs of medical care for CHIKV were estimated at EUR 12.4 million (approximately USD 13.3 million), with EUR 5 million in terms of drugs (approximately USD 5.38 million), while the cost of hospitalization was estimated at EUR 8.5 million (approximately USD 9.15 million). Including direct and indirect costs, the estimated total cost was EUR 43.9 million (USD 47.5 million) [86].

Another study in Asia found that people affected by chikungunya spent approximately BDT 8192 per person (USD 76.18) [42]. In the Americas, the adult population of Colombia faced a direct medical cost of USD 66.6 (USD 26.5 to 317.3), with a higher value in the search for specialist doctors (57.4% of expenses) [88]. Another study in the same region, carried out by Cardona-Ospina et al. in 2014, calculated a cost ranging from USD 73.6 million (most conservative scenario) to USD 185.5 million (worst scenario). Of this, the cost per patient would be between USD 1438 and 3396 in the first year of the disease, but the chronic phase increased this value by up to 95% due to the required medicines, especially effect-modifying drugs [13].

The outbreak recorded in the Virgin Islands (USA) in 2014–2015 cost more than USD 2.9 million in medical consultations alone. The direct and indirect costs of this outbreak have been estimated to range from USD 14,827,500 to 33,424,600 [91]. In Brazil, the estimate of indirect expenses for the CHIKV epidemic, which occurred in the same period, was BRL 123,943,728 (approximately USD 24.91 million) [92].

Few reports have been published on the pediatric cost of epidemics caused by CHIKV. The study of Alvis-Zakzuk et al. in Colombia estimated the expenditure at USD 257.9 (USD 121.7 to 563.8). The largest portion of the pediatric cost was associated with the cost of hospital beds (40.0%), followed by 36.4% associated with diagnostic procedures [88].

## 4. Discussion

Interestingly, in addition to the standard descriptions of epidemics and symptoms, many authors tried to address long-term sequelae and how people’s lives were affected during the acute and chronic phases of the illness. In our review, only a few studies addressed hospitalization or severe cases; most were focused on the African continent, where sequelae were considered to be significant, and atypical findings were described with more interest (such as severe and hospitalized CHIKV cases). These articles supported the notion that CHIKV, rather than an acute nonfatal disease, is a disease that can progress in severity, exhibit atypical findings, and have long-term consequences and fatal outcomes.

CHIKV is widely distributed globally, mainly in Africa, Asia, South America, and Oceania/the Pacific Islands [82]. The highest prevalence in this study was observed in Bangladesh (80%), Thailand (71%), and some African countries (70%) [5,24,41]. Despite some studies revealing an increased prevalence of chikungunya over time in certain countries, such as Tanzania [24,34,35] and Fiji [47], a meta-analysis revealed a drop worldwide over time when comparing 2000–2009 and 2010–2019 [82]. In some African regions, CHIKV was more broadly diffused in certain territories than other arboviruses such as DENV [24]. Additionally, CHIKV had a broader distribution in some regions of South America, even affecting continental countries such as Brazil, and coexisted with arboviruses such as DENV, ZIKV, and YFV [79]. Our epidemiological analysis confirmed the capacity of CHIKV to cause epidemics and rapidly spread through countries. As illustrated by the Reunion Islands [87], many countries that had never encountered a case of chikungunya were still vulnerable to the devastating impact of the disease, which, despite an acute fever, can generate long-term losses due to its chronicity.

Several studies evaluated the clinical aspects of CHIKV, emphasizing symptom prevalence, complications, chronicity, and severity. According to the literature, fever and arthralgia remain the most common symptoms [24,25,26,35,37,40,41,42,43,45,47,49,52,64,65,83]. Other complications are rare but do appear—mainly neurological complications [35,37,85]. About 13.66% of cases may evolve into CCA [84]. Several articles evaluated the risks of chronicity and found that older age [36,37,38,39,40,41,42,43,44,45,46,47,48,49,50,51,52,53,54,55,56,57,58,59,60,61,62,63,64,65,66,67,68,69,70,71,72,73,74,75,76,77,78,79,80,81,82,83,84,85,86,87,88,89,90,91,92] and duration of acute symptoms [53] were the main risk factors. Few studies have assessed the severity and hospitalization rates of chikungunya [42,44,48] or the risks associated with increasing severity [83]. Increased age and the presence of comorbidities were principal factors in severity, hospitalization, and mortality in these patients [39,81].

However, the burden of CHIKV infection is not only related to chronicity, as evidenced by its excess mortality, challenging the paradigm of a non-fatal disease [2]. As a result, CHIKV is increasingly understood as a disease that, in addition to developing chronicity and impacting life in the long term, generating absenteeism and losses, can also increase the mortality rate of the affected population in epidemic situations. Fatal outcomes occur mainly in high-risk patients with comorbidities and at the extremes of age. Chikungunya symptoms overlap with those of DENV. The clinical spectrum of CHIKV infection is wide and includes life-threatening manifestations affecting several organs. Importantly, acute dengue infection tends to present an early severity and mortality, while complicated CHIKV infection can occur during both the acute illness phase and weeks/months later, in relation to the decompensation of comorbidities such as diabetes [93]. CHIKV-related mortality is still the subject of scientific debate. However, several articles presented in this review, mainly from South America and involving northeastern Brazil, revealed high mortality rates not necessarily attributable to excess mortality alone [66,81]. Further and detailed postmortem studies might be needed to understand CHIKV-associated mortality in more depth.

The burden of other arboviruses has also been studied. Well-known viruses such as DENV and ZIKV have an associated burden related to deaths and neonatal involvement. Fernandes et al. recently published an article about the burden of ZIKV. They identified the main problem as the impact of congenital Zika syndrome (CZS) in relation to the societal cost per child [94]. Nevertheless, although sporadic cases of vertical transmission have been described, congenital and neonatal CHIKV infection appears to be very rare [95]. 

Our study had several limitations. Despite the valuable evidence provided, a meta-analysis could not be performed. The cost articles were difficult to analyze because they used multiple methodologies. Our methodology did not consider the gray literature, though sources other than academic journals may offer valuable information that could have been missed. 

This study provided an overview of the burden of CHIKV worldwide, addressing issues related to epidemiology, economic costs, and mortality. An economic perspective was presented separately from the epidemiological perspective. We hope that this study will be considered by policymakers, especially when prioritizing the allocation of health resources and preparing disease prevention programs. The health and economic burden of CHIKV is more important than initially thought. Further research on the impact of the disease, studies to assess CHIKV-related mortality more thoroughly, and the reinforcement of surveillance programs are needed.

## 5. Conclusions

With more than 100 countries having already experienced CHIKV epidemics, the virus continues to represent a risk to many others, which must be prepared to face the emergence of CHIKV. Countries unfamiliar with the disease should be aware of the emergent potential of CHIKV. The burden of the CCA disease associated with CHIKV infection is highly significant worldwide. CHIKV’s burden is associated with chronicity, severity, hospitalization risks, and mortality. Understanding and measuring the full impact of chikungunya as a clinical disease that affects more than just individual patients is essential. Chikungunya can generate economic losses in several spheres, significantly affecting the health system and national economies. This article provides a starting point for a more comprehensive discussion of the global impact of this disease. These data will help guide improvements in disease control strategies employing vaccines, medications, and vector control techniques, as well as their economic evaluation.

## Figures and Tables

**Table 1:** Economic impacts of chikungunya on different continents.

Country/Region	Direct Costs (USD)	Loss in Productivity (USD)	DALYs
India/Asia	16.680	2.57–4.69 million	25,588
Reunion Islands/French Department	36.72 million	18.79 million	-
Colombia/South America	121.7–563.8 per patient	72.2–203.2 per patient	350,531
Brazil/South America	-	2.13 billion	0.036
Mexico/North America	-	130 thousand	-
USA/North America	2.9 million	-	-

Note: values converted from local currency to USD on 18 May 2023.

## Data Availability

All data supporting the findings of this study are available in Supplementary Materials.

## Supplementary Materials

The following supporting information can be downloaded at: https://www.mdpi.com/article/10.3390/tropicalmed8060301/s1. Table S1: Bibliographic search strategy, Table S2: Data extraction and main characteristics of the included studies, Table S3: Quality assessment of included studies.

## References

[1] Doran C., Elsinga J., Fokkema A., Berenschot K., Gerstenbluth I., Duits A., Lourents N., Halabi Y., Burgerhof J., Bailey A. (2022). Long-term Chikungunya sequelae and quality of life 2.5 years post-acute disease in a prospective cohort in Curaçao. PLoS Negl. Trop. Dis..

[2] Freitas A.R.R., Alarcón-Elbal P.M., Donalisio M.R. (2018). Excess mortality in Guadeloupe and Martinique, islands of the French West Indies, during the chikungunya epidemic of 2014. Epidemiol. Infect..

[3] Frutuoso L.C.V., Freitas A.R.R., Cavalcanti L., Duarte E.C. (2020). Estimated mortality rate and leading causes of death among individuals with chikungunya in 2016 and 2017 in Brazil. Rev. Soc. Bras. Med. Trop..

[4] Kumar R., Ahmed S., Parray H.A., Das S. (2021). Chikungunya and arthritis: An overview. Travel Med. Infect. Dis..

[5] Khongwichit S., Chansaenroj J., Chirathaworn C., Poovorawan Y. (2021). Chikungunya virus infection: Molecular bi-ology, clinical characteristics, and epidemiology in Asian countries. J. Biomed. Sci..

[6] Manzoor K.N., Javed F., Ejaz M., Ali M., Mujaddadi N., Khan A.A., Khattak A.A., Zaib A., Ahmad I., Saeed W.K. (2022). The global emergence of Chikungunya infection: An integrated view. Rev. Med. Virol..

[7] Matusali G., Colavita F., Bordi L., Lalle E., Ippolito G., Capobianchi M.R., Castilletti C. (2019). Tropism of the chikungunya virus. Viruses.

[8] Robinson M.C. (1955). An epidemic of virus disease in Southern Province, Tanganyika territory, in 1952–1953. Trans. R. Soc. Trop. Med. Hyg..

[9] Ross R.W. (1956). The Newala epidemic: III. The virus: Isolation, pathogenic properties and relationship to the epi-demic. J. Hyg..

[10] Wahid B., Ali A., Rafique S., Idrees M. (2017). Global expansion of chikungunya virus: Mapping the 64-year history. Int. J. Infect. Dis..

[11] Grandadam M., Caro V., Plumet S., Thiberge J.M., Souarès Y., Failloux A.B., Tolou H.J., Budelot M., Cosserat D., Leparc-Goffart I. (2011). Chikungunya virus, Southeastern France. Emerg. Infect. Dis..

[12] Rezza G., Nicoletti L., Angelini R., Romi R., Finarelli A.C., Panning M., Cordioli P., Fortuna C., Boros S., Magurano F. (2007). Faculty Opinions recommendation of Infection with chikungunya virus in Italy: An outbreak in a temperate region. Lancet.

[13] Cardona-Ospina J.A., Henao-SanMartin V., Paniz-Mondolfi A.E., Rodríguez-Morales A.J. (2015). Mortality and fatality due to Chikungunya virus infection in Colombia. J. Clin. Virol..

[14] Cardona-Ospina J.A., Diaz-Quijano F.A., Rodríguez-Morales A.J. (2015). Burden of chikungunya in Latin American countries: Estimates of disability-adjusted life-years (DALY) lost in the 2014 epidemic. Int. J. Infect. Dis..

[15] Lindh E., Argentini C., Remoli M.E., Fortuna C., Faggioni G., Benedetti E., Amendola A., Marsili G., Lista F., Rezza G. (2019). The Italian 2017 outbreak chikungunya virus belongs to an emerging aedes albopictus-adapted virus cluster introduced from the Indian sub-continent. Open Forum Infectious Diseases.

[16] Higgins J.P., Thomas J., Chandler J., Cumpston M., Li T., Page M.J. (2019). Cochrane Handbook for Systematic Reviews of Interventions.

[17] Page M.J., McKenzie J.E., Bossuyt P.M., Boutron I., Hoffmann T.C., Mulrow C.D., Shamseer L., Tetzlaff J.M., Akl E.A., Brennan S.E. (2021). The PRISMA 2020 Statement: An Updated Guideline for Reporting Systematic Reviews. BMJ.

[18] Ma L.-L., Wang Y.-Y., Yang Z.-H., Huang D., Weng H., Zeng X.-T. (2020). Methodological quality (risk of bias) assessment tools for primary and secondary medical studies: What are they and which is better?. Mil. Med. Res..

[19] Wells G.A., Shea B., O’Connell D., Peterson J., Welch V., Losos M., Tugwell P. (2000). The Newcastle-Ottawa Scale (NOS) for Assessing the Quality of Nonrandomised Studies in Meta-Analyses. https://www.ohri.ca/programs/clinical_epidemiology/oxford.asp.

[20] Munn Z., Moola S., Lisy K., Riitano D., Tufanaru C. (2015). Methodological guidance for systematic reviews of observa-tional epidemiological studies reporting prevalence and cumulative incidence data. Int. J. Evid.-Based Healthc..

[21] Silva EN D., Silva M.T., Augustovski F., Husereau D., Pereira M.G. (2017). Roteiro para relato de es-tudos de avaliação econômica. Epidemiol. Serviços Saúde.

[22] Shea B.J., Reeves B.C., Wells G., Thuku M., Hamel C., Moran J., Moher D., Tugwell P., Welch V., Kristjansson E. (2017). AMSTAR 2: A critical appraisal tool for systematic reviews that include randomised or non-randomised studies of healthcare interventions, or both. BMJ.

[23] Renault P., Solet J.L., Sissoko D., Balleydier E., Larrieu S., Filleul L., Lassalle C., Thiria J., Rachou E., de Valk H. (2007). A major epidemic of chikungunya virus infection on Réunion Island, France, 2005–2006. Am. J. Trop. Med. Hyg..

[24] Mwanyika G.O., Sindato C., Rugarabamu S., Rumisha S.F., Karimuribo E.D., Misinzo G., Rweyemamu M.M., Hamid M.M.A., Haider N., Vairo F. (2021). Seroprevalence and associated risk factors of chikungunya, dengue, and Zika in eight districts in Tanzania. Int. J. Infect. Dis..

[25] Sissoko D., Moendandzé A., Malvy D., Giry C., Ezzedine K., Solet J.L., Pierre V. (2008). Seroprevalence and Risk Factors of Chikungunya Virus Infection in Mayotte, Indian Ocean, 2005–2006: A Population-Based Survey. PLoS ONE.

[26] Sergon K., Sang R., Brown J., Onyango C., Powers A.M., Agata N., Njenga M.K., Bedja S.A., Allaranger Y., Konongoi L.S. (2007). Seroprevalence of Chikungunya virus infection on Grande Comore Island, Union of the Comoros, 2005. Am. J. Trop. Med. Hyg..

[27] Sergon K., Njuguna C., Kalani R., Ofula V., Onyango C., Konongoi L.S., Bedno S., Burke H., Dumilla A.M., Konde J. (2008). Seroprevalence of Chikungunya virus (CHIKV) infection on Lamu Island, Kenya, October 2004. Am. J. Trop. Med. Hyg..

[28] Mease L.E., Coldren R.L., Musila L.A., Prosser T., Ogolla F., Ofula V.O., Schoepp R.J., Rossi C.A., Adungo N. (2011). Seroprevalence and distribution of ar-boviral infections among rural Kenyan adults: A cross-sectional study. Virol. J..

[29] Moyen N., Thiberville S.-D., Pastorino B., Nougairede A., Thirion L., Mombouli J.-V., Dimi Y., Leparc-Goffart I., Capobianchi M.R., Lepfoundzou A.D. (2014). First Reported Chikungunya Fever Outbreak in the Republic of Congo, 2011. PLoS ONE.

[30] Endale A., Michlmayr D., Abegaz W.E., Asebe G., Larrick J.W., Medhin G., Legesse M. (2020). Community-based sero-prevalence of chikungunya and yellow fever in the South Omo Valley of Southern Ethiopia. PLOS Negl. Trop. Dis..

[31] António V.S., Muianga A.F., Wieseler J., Pereira S.A., Monteiro V.O., Mula F., Chelene I., Chongo I.S., Oludele J.O., Kümmerer B.M. (2018). Seroepidemiology of Chikungunya Virus among Febrile Patients in Eight Health Facilities in Central and Northern Mozambique, 2015–2016. Vector-Borne Zoonotic Dis..

[32] Abdullahi I.N., Akande A.O., Muhammed Y., Rogo L.D., Oderinde B. (2020). Prevalence Pattern of Chikungunya Virus Infection in Nigeria: A Four Decade Systematic Review and Meta-analysis. Pathog. Glob. Health.

[33] Ekong P.S., Aworh M.K., Grossi-Soyster E.N., Wungak Y.S., Maurice N.A., Altamirano J., Ekong M.J., Olugasa B.O., Nwosuh C.I., Shamaki D. (2022). A Retrospective Study of the Seroprevalence of Dengue Virus and Chikungunya Virus Exposures in Nigeria, 2010–2018. Pathogens.

[34] Hertz J.T., Munishi O.M., Ooi E.E., Howe S., Lim W.Y., Chow A., Morrissey A.B., Bartlett J.A., Onyango J.J., Maro V.P. (2012). Chikungunya and dengue fever among hospitalized febrile patients in northern Tanzania. Am. J. Trop. Med. Hyg..

[35] Kinimi E., Shayo M.J., Patrick B.N., Angwenyi S.O., Kasanga C.J., Weyer J., Jansen van Vuren P., Paweska J.T., Mboera L.E., Misinzo G. (2018). Evidence of chikungunya virus infection among febrile patients seeking healthcare in selected districts of Tanzania. Infect. Ecol. Epidemiol..

[36] Sergon K., Njuguna C., Kalani R., Ofula V., Onyango C., Konongoi L.S., Bedno S., Burke H., Dumilla A.M., Konde J. (2013). Chikungunya Virus-associated Long-term Arthralgia: A 36-month Prospective Longitudinal Study. PLoS Negl. Trop. Dis..

[37] Soumahoro M.K., Gerardin P., Boelle P.Y., Perrau J., Fianu A., Pouchot J., Malvy D., Flahault A., Favier F., Hanslik T. (2009). Impact of Chikungunya Virus Infection on Health Status and Quality of Life: A Retrospective Cohort Study. Klein R, editor. PLoS ONE.

[38] Gérardin P., Fianu A., Malvy D., Mussard C., Boussaïd K., Rollot O., Michault A., Gaüzere B.A., Bréart G., Favier F. (2011). Perceived morbidity and community burden after a Chikungunya outbreak: The TELECHIK survey, a population-based cohort study. BMC Med..

[39] Economopoulou A., Dominguez M., Helynck B., Sissoko D., Wichmann O., Quenel P., Germonneau P., Quatresous I. (2009). Atypical Chikungunya virus infections: Clinical manifestations, mortality and risk factors for severe disease during the 2005–2006 outbreak on Réunion. Epidemiol. Infect..

[40] Azami N.A.M., Salleh S.A., Shah S.A., Neoh H.M., Othman Z., Zakaria S.Z.S., Jamal R. (2013). Emergence of chikungunya seropositivity in healthy Malaysian adults residing in outbreak-free locations: Chikungunya seroprevalence results from the Malaysian Cohort. BMC Infect. Dis..

[41] Khatun S., Chakraborty A., Rahman M., Nasreen Banu N., Rahman M.M., Hasan S.M., Luby S.P., Gurley E.S. (2015). An outbreak of chikungunya in rural Bangladesh, 2011. PLoS Negl. Trop. Dis..

[42] Hossain M.S., Hasan M.M., Islam M.S., Islam S., Mozaffor M., Khan M.A.S., Ahmed N., Akhtar W., Chowdhury S., Arafat S.Y. (2018). Chikungunya outbreak (2017) in Bangladesh: Clinical profile, economic impact and quality of life during the acute phase of the disease. PLoS Negl. Trop. Dis..

[43] Ray P., Ratagiri V.H., Kabra S.K., Lodha R., Sharma S., Sharma B.S., Kalaivani M., Wig N. (2012). Chikungunya infection in India: Results of a prospective hospital based multi-centric study. PLoS ONE.

[44] Chattopadhyay S., Mukherjee R., Nandi A., Bhattacharya N. (2016). Chikungunya virus infection in West Bengal, India. Indian J. Med. Microbiol..

[45] Kumar M.S., Kamaraj P., Khan S.A., Allam R.R., Barde P.V., Dwibedi B., Kanungo S., Mohan U., Mohanty S.S., Roy S. (2021). Seroprevalence of chikungunya virus infection in India, 2017: A cross-sectional population-based serosurvey. Lancet Microbe.

[46] Khongwichit S., Chansaenroj J., Thongmee T., Benjamanukul S., Wanlapakorn N., Chirathaworn C., Poovorawan Y. (2021). Large-scale outbreak of Chikungunya virus infection in Thailand, 2018–2019. PLoS ONE.

[47] Aubry M., Kama M., Henderson A.D., Teissier A., Vanhomwegen J., Mariteragi-Helle T., Paoaafaite T., Manuguerra J.C., Christi K., Watson C.H. (2020). Low chikungunya virus seroprevalence two years after emergence in Fiji. Int. J. Infect. Dis..

[48] Dutta P., Khan S.A., Phukan A.C., Hazarika S., Hazarika N.K., Chetry S., Khan A.M., Kaur H. (2019). Surveillance of Chikungunya virus activity in some North-eastern states of India. Asian Pac. J. Trop. Med..

[49] Barr K.L., Khan E., Farooqi J.Q., Imtiaz K., Prakoso D., Malik F., Lednicky J.A., Long M.T. (2018). Evidence of Chikungunya Virus Disease in Pakistan Since 2015 With Patients Demonstrating Involvement of the Central Nervous System. Front. Public Health.

[50] Badar N., Ikram A., Salman M., Alam M.M., Umair M., Arshad Y., Mushtaq N., Mirza H.A., Ahad A., Yasin M.T. (2021). Epidemiology of Chikungunya virus isolates 2016–2018 in Pakistan. J. Med. Virol..

[51] Joshi P., Yadav P., Mourya D., Sahare L., Ukey M., Khedekar R., Patil D., Barde P.V. (2020). Laboratory surveillance of chikungunya in Madhya Pradesh, India (2016–2017). Indian J. Med. Res..

[52] Mathew A.J., Goyal V., George E., Thekkemuriyil D.V., Jayakumar B., Chopra A. (2011). Rheumatic-musculoskeletal pain and disorders in a naïve group of individuals 15 months following a Chikungunya viral epidemic in south India: A population based observational study. Int. J. Clin. Pract..

[53] Ramachandran V., Malaisamy M., Ponnaiah M., Kaliaperuaml K., Vadivoo S., Gupte M.D. (2012). Impact of Chikungunya on health related quality of life Chennai, South India. PLoS ONE.

[54] Vongpunsawad S., Intharasongkroh D., Thongmee T., Poovorawan Y. (2017). Seroprevalence of antibodies to dengue and chikungunya viruses in Thailand. PLoS ONE.

[55] Murhekar M., Kanagasabai K., Shete V., Joshua V., Ravi M., Kirubakaran B.K., Ramachandran R., Sabarinathan R., Gupta N. (2019). Epidemiology of chikungunya based on laboratory surveillance data—India, 2016–2018. Trans. R. Soc. Trop. Med. Hyg..

[56] Luvai E.A.C., Kyaw A.K., Sabin N.S., Yu F., Hmone S.W., Thant K.Z., Inoue S., Morita K., Ngwe Tun M.M. (2021). Evidence of Chikungunya virus seroprevalence in Myanmar among denguesuspected patients and healthy volunteers in 2013, 2015, and 2018. PLoS Negl. Trop. Dis..

[57] Chopra A., Ghorpade R., Venugopalan A., Saluja M., Adam K. (2020). Increased Burden of Painful Arthritis and Rheumatism Following the Chikungunya Epidemic 2006: India Rural Population Survey 2018. Arthritis Rheumatol..

[58] Henry M., Francis L., Asin V., Polson-Edwards K., Olowokure B. (2017). Chikungunya virus outbreak in Sint Maarten, 2013–2014. Rev. Panam. Salud Publica.

[59] Couzigou B., Criquet-Hayot A., Javelle E., Tignac S., Mota E., Rigaud F., Alain A., Troisgros O., Pierre-Francois S., Abel S. (2018). Occurrence of Chronic Stage Chikungunya in the General Population of Martinique during the First 2014 Epidemic: A Prospective Epidemiological Study. Am. J. Trop. Med. Hyg..

[60] Gallian P., Leparc-Goffart I., Richard P., Maire F., Flusin O., Djoudi R., Chiaroni J., Charrel R., Tiberghien P., de Lamballerie X. (2017). Epidemiology of Chikungunya Virus Outbreaks in Guadeloupe and Martinique, 2014: An Observational Study in Volunteer Blood Donors. PLoS Negl. Trop. Dis..

[61] Hennessey M.J., Ellis E.M., Delorey M.J., Panella A.J., Kosoy O.I., Kirking H.L., Appiah G.D., Qin J., Basile A.J., Feldstein L.R. (2018). Seroprevalence and symptomatic attack rate of chikungunya virus infection, United States virgin islands, 2014–2015. Am. J. Trop. Med. Hyg..

[62] Sharp T.M., Ryff K.R., Alvarado L., Shieh W.J., Zaki S.R., Margolis H.S., Rivera-Garcia B. (2016). Surveillance for chikungunya and dengue during the first year of chikungunya virus circulation in puerto rico. J. Infect. Dis..

[63] Ministerio del Poder Ciudadano para la Salud de Nicaragua (2017). Seroprevalencia y tasa de ataque clínica por chikungunya en Nicaragua, 2014–2015. Rev. Panam. Salud Pública.

[64] Gordon A., Gresh L., Ojeda S., Chowell G., Gonzalez K., Sanchez N., Saborio S., Mercado J.C., Kuan G., Balmaseda A. (2018). Differences in Transmission and Disease Severity between 2 Successive Waves of Chikungunya. Clin. Infect. Dis..

[65] Dorléans F., Hoen B., Najioullah F., Herrmann-Storck C., Schepers K.M., Abel S., Lamaury I., Fagour L., Cesaire R., Guyomard S. (2018). Outbreak of chikungunya in the French caribbean islands of martinique and guadeloupe: Findings from a hospital-Based surveillance system (2013–2015). Am. J. Trop. Med. Hyg..

[66] Crosby L., Perreau C., Madeux B., Cossic J., Armand C., Herrmann-Storke C., Najioullah F., Valentino R., Thiéry G. (2016). Severe manifestations of chikungunya virus in critically ill patients during the 2013–2014 Caribbean outbreak. Int. J. Infect. Dis..

[67] Crosby L., Perreau C., Madeux B., Cossic J., Armand C., Herrmann-Storke C., Najioullah F., Valentino R., Thiéry G. (2020). Prevalence of chronic chikungunya and associated risks factors in the French West Indies (La Martinique): A prospective cohort study. PLoS Negl. Trop. Dis..

[68] Huits R., De Kort J., Berg R.V.D., Chong L., Tsoumanis A., Eggermont K., Bartholomeeusen K., Arien K.K., Jacobs J., Van Esbroeck M. (2018). Chikungunya virus infection in Aruba: Diagnosis, clinical features and predictors of post-chikungunya chronic polyarthralgia. PloS ONE.

[69] Mercado-Reyes M., Acosta-Reyes J., Navarro-Lechuga E., Corchuelo S., Rico A., Parra E., Tolosa N., Pardo L., González M., Martìn-Rodriguez-Hernández J. (2019). Dengue, chikungunya and zika virus coinfection: Results of the national surveillance during the zika epidemic in Colombia. Epidemiol. Infect..

[70] Ster I.C., Rodriguez A., Romero N.C., Lopez A., Chico M., Montgomery J., Cooper P. (2020). Age-dependent seroprevalence of dengue and chikungunya: Inference from a cross-sectional analysis in Esmeraldas Province in coastal Ecuador. BMJ Open.

[71] Bailly S., Rousset D., Fritzell C., Hozé N., Ben Achour S., Berthelot L., Enfissi A., Vanhomwegen J., Salje H., Fernandes-Pellerin S. (2021). Spatial distribution and burden of emerging arboviruses in French Guiana. Viruses.

[72] Dias J.P., Maria da Conceição N.C., Campos G.S., Paixão E.S., Natividade M.S., Barreto F.R., Itaparica M.S.C., Goes C., Oliveira F.L., Santana E.B. (2018). Seroprevalence of Chikungunya Virus after Its Emergence in Brazil. Emerg. Infect. Dis..

[73] Cunha R.V., Trinta K.S., Montalbano C.A., Sucupira M.V.F., de Lima M.M., Marques E., Romanholi I.H., Croda J. (2017). Seroprevalence of Chikungunya Virus in a Rural Community in Brazil. PLOS Neglected Trop. Dis..

[74] Teixeira M.G., Skalinski L.M., Paixão E.S., Costa M.D.C.N., Barreto F.R., Campos G.S., Sardi S.I., Carvalho R.H., Natividade M., Itaparica M. (2021). Seroprevalence of Chikungunya virus and living conditions in Feira de Santana, Bahia-Brazil. PLOS Negl. Trop. Dis..

[75] Nicacio J.M., Khouri R., da Silva A.M.L., Barral-Netto M., Lima J.A.C., Ladeia A.M.T., Carmo R.F.D., Armstrong A.D.C. (2021). Anti-chikungunya virus seroprevalence in Indigenous groups in the São Francisco Valley, Brazil. PLOS Negl. Trop. Dis..

[76] Anjos R.O., Mugabe V.A., Moreira P.S., Carvalho C.X., Portilho M.M., Khouri R., Sacramento G.A., Nery N.R., Reis M.G., Kitron U.D. (2020). Transmission of Chikungunya Virus in an Urban Slum, Brazil. Emerg. Infect. Dis..

[77] Simião A.R., Barreto F.K.D.A., Oliveira R.D.M.A.B., Cavalcante J.W., Neto A.S.L., Barbosa R.B., Lins C.D.S., Meira A.G., Araújo F.M.D.C., Lemos D.R.Q. (2019). A major chikungunya epidemic with high mortality in northeastern Brazil. Rev. Soc. Bras. Med. Trop..

[78] Barreto M.C.A., Gomes I.P., de Castro S.S. (2021). Qualidade de vida dos pacientes com chikungunya: Fatores associados durante uma epidemia ocorrida no nordeste do Brasil. J. Health Biol. Sci..

[79] Barreto F.K.A., Alencar C.H., Araújo F.M.D.C., Oliveira R.D.M.A.B., Cavalcante J.W., Lemos D.R.Q., Farias L.A.B.G., Boriz I.L.F., Medeiros L.Q., Melo M.N.P. (2020). Seroprevalence, spatial dispersion and factors associated with flavivirus and chikungunya infection in a risk area: A population-based seroprevalence study in Brazil. BMC Infect. Dis..

[80] Périssé A.R.S., Souza-Santos R., Duarte R., Santos F., De Andrade C.R., Rodrigues N.C.P., Schramm J.M.D.A., Da Silva E.D., Jacobson L.D.S.V., Lemos M.C.F. (2020). Zika, dengue and chikungunya population prevalence in Rio de Janeiro city, Brazil, and the importance of seroprevalence studies to estimate the real number of infected individuals. PLoS ONE.

[81] Vidal E.R.N., Frutuoso L.C.V., Duarte E.C., Peixoto H.M. (2021). Epidemiological burden of Chikungunya fever in Brazil, 2016 and 2017. Trop. Med. Int. Health.

[82] Li Z., Wang J., Cheng X., Hu H., Guo C., Huang J., Chen Z., Lu J. (2021). The worldwide seroprevalence of DENV, CHIKV and ZIKV infection: A systematic review and meta-analysis. PloS Negl. Trop. Dis..

[83] Badawi A., Ryoo S.G., Vasileva D., Yaghoubi S. (2018). Prevalence of chronic comorbidities in chikungunya: A system-atic review and meta-analysis. Int. J. Infect. Dis..

[84] Rodríguez-Morales A.J., Cardona-Ospina J.A., Fernanda Urbano-Garzón S., Sebastian Hurtado-Zapata J. (2016). Preva-lence of Post-Chikungunya Infection Chronic Inflammatory Arthritis: A Systematic Review and Meta-Analysis. Arthritis Care Res..

[85] Krishnamoorthy K., Harichandrakumar K.T., Kumari A.K., Das L.K. (2009). Burden of chikungunya in India: Estimates of disability adjusted life years (DALY) lost in 2006 epidemic. J. Vector Borne Dis..

[86] Gopalan S.S., Das A. (2009). Household economic impact of an emerging disease in terms of catastrophic out-of-pocket health care expenditure and loss of productivity: Investigation of an outbreak of chikungunya in Orissa, India. J. Vector Borne Dis..

[87] Soumahoro M.-K., Boelle P.-Y., Gaüzere B.-A., Atsou K., Pelat C., Lambert B., La Ruche G., Gastellu-Etchegorry M., Renault P., Sarazin M. (2011). The Chikungunya Epidemic on La Réunion Island in 2005–2006: A Cost-of-Illness Study. PLOS Negl. Trop. Dis..

[88] Alvis-Zakzuk N.J., Díaz-Jiménez D., Castillo-Rodríguez L., Castañeda-Orjuela C., Paternina-Caicedo Á., Pinzón-Redondo H., Carrasquilla-Sotomayor M., Alvis-Guzmán N., De La Hoz-Restrepo F. (2018). Economic Costs of Chikungunya Virus in Colombia. Value Health Reg. Issues.

[89] Vázquez-Cruz I., Juanico-Morales G., Sanchez-Ramos A., de Jesús Morales-Sánchez O. (2018). Costs and sick leave due to chikungunya in the Instituto Mexicano del Seguro Social in Guerrero, Mexico. Rev. Médica Del Inst. Mex. Del Seguro Soc..

[90] Feldstein L.R., Ellis E.M., Rowhani-Rahbar A., Hennessey M.J., Staples J.E., Halloran M.E., Weaver M.R. (2019). Estimating the cost of illness and burden of disease associated with the 2014–2015 chikungunya outbreak in the US Virgin Islands. PloS Negl. Trop. Dis..

[91] Mora-Salamanca A.F., Porras-Ramírez A., Restrepo F.P.D.L.H. (2020). Estimating the burden of arboviral diseases in Colombia between 2013 and 2016. Int. J. Infect. Dis..

[92] Teich V., Arinelli R., Fahham L. (2017). Aedes aegypti e sociedade: O impacto econômico das rboviruses no Brasil. J. Bras. Econ. Saúde.

[93] De Almeida Barreto F.K., Montenegro R.M., Fernandes V.O., Oliveira R., de Araújo Batista L.A., Hussain A., de Góes Cavalcanti L.P. (2018). Chikungunya and diabetes, what do we know?. Diabetol. Metab. Syndr..

[94] Fernandes S., Pinto M., Barros L., Moreira M.E.L., de Araújo T.V.B., Lyra T.M., Valongueiro S., Jofre-Bonet M., Kuper H. (2022). The economic burden of congenital Zika Syndrome in Brazil: An overview at 5 years and 10 years. BMJ Glob. Health.

[95] Gopakumar H., Ramachandran S. (2012). Congenital chikungunya. J. Clin. Neonatol..

