# Supplementary material for: Epidemiology and Economic Burden of Chikungunya: A Systematic Literature Review
Source: Trop Med Infect Dis. 2023 May 31;8(6):301. doi: 10.3390/tropicalmed8060301 (PMC10302198; doi:10.3390/tropicalmed8060301)
Supplement: Supplementary file 1 [file tropicalmed-08-00301-s001.zip › SM_1_SEARCH_STRATEGY.pdf]

PLANNING SYSTEMATIC REVIEW CHIKUNGUNYA COSTS

1. **Review Guiding Question**

What is the epidemiological and economic burden of chikungunya in the world?

	**Inclusion Criteria**	**Exclusion criteria**
**Population**	Individuals of all ages with chikungunya or prior exposure to chikungunya	Studies not reporting patients with chikungunya or prior exposure to chikungunya
Intervention	*SLR not restricted by intervention criteria*	
Comparison	*SLR not restricted by comparison criteria*	
**Outcome**	Epidemiological data of chikungunya, such as:<br>- Prevalence<br>- Incidence<br>- Seroprevalence<br>- Studies that include information on the severity of chikungunya (morbidity, mortality, hospitalization)	Studies published outside date limits: 2007-2022
	Cost of illness and economic burden of chikungunya for patients and health services and/or social perspective, such as:<br>- Direct medical costs<br>- Direct non-medical costs<br>- Indirect social costs	
**Study design**	● Randomized studies<br>● Non-randomized controlled study designs, including:<br>○ Non-randomized controlled trial.<br>○ Cohort studies.<br>○ Case control studies.	● Publications that do not clearly describe methods and sources for data collection/analysis<br>● In vitro study<br>● Cost-effective models<br>● Clinical trials that do not report

	○ Cross-sectional studies.<br>● Outbreak reports.<br>● Genomic studies.<br>● Systematic literature reviews if a meta-analysis is included.<br>● Cost of illness or burden of illness studies.	baseline/or look at the placebo/control group in the results<br>● News and opinion articles.<br>Studies focusing exclusively on imported-cases of CHIKV.

1. **Estratégia de busca** (guia https://www.cadth.ca/press-peer-review-electronic-search-strategies-0)**:**

Systematic search strategy serves to avoid a selective choice. Definition of text-words and descriptions – PICO. Search filter – T-T. Use of Boolean operators – AND/OR/NOT.

**1**	Chickungunya OR Chickungunya Fever OR Chickungunya Fevers OR Chikungunya Fevers OR Chikungunya Virus Infection OR Chikungunya Virus Infections	Febre de Chikungunya OR Chicungunha OR Chikungunya OR Febre Chickungunya OR Febre Chikungunya OR Febre do Chikungunya OR Infecção pelo Vírus Chikungunya OR Infecção por Vírus Chikungunya	Fiebre Chikungunya OR Chickungunya OR Chikungunya OR Chikunguña OR Fiebre Chickungunya OR Fiebre Chikunguña OR Fiebre de Chikungunya OR Fiebre de Chikunguña OR Fiebre por el Virus del Chikungunya OR Infección por el Virus Chikungunya OR Infección por Virus Chikungunya
**2**	Cross-Sectional Studies OR Cross Sectional Analyses OR Cross Sectional Analysis OR Cross Sectional Studies OR Cross Sectional Survey OR Cross-Sectional Analyses OR Cross-Sectional Analysis	Estudos Transversais OR Estudos de Corte Transversal OR Estudos de Prevalência OR Estudos Seccionais OR Levantamentos de Frequência de Doenças	Estudios Transversales OR Encuestas de Frencuencia de Enfermedades OR Estudios de Corte Transversal OR Estudios de Prevalencia OR Estudios Seccionales

	OR Cross-Sectional Study OR Cross-Sectional Survey OR Cross-Sectional Surveys OR Disease Frequency Survey OR Disease Frequency Surveys OR Prevalence Studies OR Prevalence Study		
**3**	Cohort Studies OR Cohort Analyses OR Cohort Analysis OR Historical Cohort Studies OR Historical Cohort Study OR Incidence Studies OR Incidence Study	Estudos de Coortes OR Análise de Coortes OR Estudos de Incidência OR Estudos Históricos de Coortes	Estudios de Cohortes OR Análisis de Cohortes OR Estudios de Incidencia OR Estudios Históricos de Cohortes
**4**	Morbidity OR Morbidities	Morbidade OR Frequência OR Morbidez OR Morbilidade OR Taxa de Morbidade	Morbidad
**5**	Mortality OR Case Fatality Rate OR Case Fatality Rates OR Crude Death Rate OR Crude Death Rates OR Crude Mortality Rate OR Crude Mortality Rates OR Death Rate OR Death Rates OR Mortalities OR Mortality Rate OR Mortality Rates	Mortalidade OR Coeficiente de Mortalidade OR Estatísticas de Mortalidade OR Índice de Casos Fatais OR Índice de Fatalidade OR Índice de Letalidade OR Índice de Mortalidade OR Letalidade OR Taxa de Casos Fatais OR Taxa de Fatalidade OR Taxa de Letalidade OR Taxa de Mortalidade	Mortalidad OR Estadísticas de Mortalidad OR Índice de Casos Fatales OR Índice de Fatalidad OR Índice de Letalidad OR Índice de Mortalidad OR Letalidad OR Tasa de Casos Fatales OR Tasa de Fatalidad OR Tasa de Letalidad OR Tasa de Mortalidad
**6**	Disability-Adjusted Life Years OR DALY OR DALYs OR Disability Adjusted Life Years OR Disability-Adjusted Life Year OR Years Lived With Disability	Anos de Vida Ajustados pela Incapacidade OR Anos de Vida Perdidos por Incapacidade OR Anos Vividos com Deficiência OR DALYs	Años de Vida Ajustados por Discapacidad OR Años de vida Ajustados por la Incapacidad OR Años de Vida Perdidos por Incapacidad OR Años Vividos con Discapacidad OR DALYs

**7**	Seroepidemiologic Studies OR Seroepidemiologic Study OR Seroepidemiological Studies OR Seroepidemiological Study OR Seroprevalence OR Seroprevalences	Estudos Soroepidemiológicos OR Soroepidemiologia OR Soroprevalência	Estudios Seroepidemiológicos OR Seroepidemiología OR Seroprevalencia
**8**	Cost of Illness OR Burden Of Disease OR Burden Of Diseases OR Burden of Illness OR Cost of Disease OR Cost of Sickness OR Disease Burden OR Disease Burdens OR Disease Cost OR Disease Costs OR Economic Burden of Disease OR Illness Burden OR Illness Burdens OR Illness Cost OR Illness Costs OR Sickness Cost OR Sickness Costs	Efeitos Psicossociais da Doença OR Carga da Doença OR Carga das Doenças OR Carga de Doença OR Custo da Doença OR Custo da Doença para o Paciente OR Custos da Doença OR Fardo da Doença OR Fardos Relativos à Doença OR Ônus da Doença OR Peso da Doença	Costo de Enfermedad OR carga de la enfermedad OR carga de las dolencias OR carga de las enfermedades OR carga de morbimortalidad OR coste de la enfermedad para el paciente OR costes de las dolencias
**9**	Cost Allocation OR Cost Allocations OR Cost Apportionment OR Cost Apportionments OR Cost Shifting OR Cost Shiftings	Alocação de Custos OR Distribuição de Custos OR Substituição de Custos	Asignación de Costos OR desviación de costes OR distribución de costes OR imputación de costes
**10**	Health Care Costs OR Health Care Cost OR Health Cost OR Health Costs OR Healthcare Cost OR Healthcare Costs OR Medical Care Cost OR Medical Care Costs OR Treatment Cost OR Treatment Costs	Custos de Cuidados de Saúde OR Custos de Cuidados Médicos OR Custos de Tratamento	costes de la atención a la salud OR costes de la asistencia sanitaria OR costes de la atención médica OR costes del tratamiento
**11**	Drug Costs OR Drug Cost	Custos de Medicamentos OR Despesa com Fármacos OR Despesa com Medicamento OR Despesa com	Costos de los Medicamentos OR coste de farmacia

		Medicamentos OR Despesas com Fármacos OR Despesas com Medicamentos OR Gasto com Fármacos OR Gasto com Medicamentos	
**12**	Direct Service Costs OR Direct Service Cost	Custos Diretos de Serviços OR Custo Direto	Costos Directos de Servicios OR Coste directo de los servicios
**13**	Hospital Costs OR Hospital Cost	Custos Hospitalares	Costos de Hospital OR costes hospitalarios
**14**	Cost Efficiency Analysis	Análise Custo-Eficiência	Análisis Costo-Eficiencia
**15**	Cost-Benefit Analysis OR Benefits and Costs OR Cost and Benefit OR Cost Benefit OR Cost Benefit Analyses OR Cost Benefit Analysis OR Cost Benefit Data OR Cost Effectiveness OR Cost Effectiveness Analysis OR Cost Utility Analysis OR Cost-Benefit Analyses OR Cost-Benefit Data OR Cost-Effectiveness Analysis OR Cost-Utility Analyses OR Cost-Utility Analysis OR Costs and Benefits OR Economic Evaluation OR Economic Evaluations OR Marginal Analyses OR Marginal Analysis OR Cost-Effectiveness Evaluation	Análise Custo-Benefício OR Análise de Custo-Benefício OR Análise de Custo-Efetividade OR Análise de Custo-Utilidade OR Análise Marginal OR Avaliação Econômica OR Custo-Benefício OR Custo-Efetividade OR Dados de Custo-Benefício OR Avaliação de Custo-Efetividade	Análisis Costo-Beneficio OR análisis de coste-utilidad OR análisis de costo-utilidad OR Coste-beneficio OR Coste-efectividad OR datos de coste-beneficio OR evaluación económica OR relación coste-eficacia
**16**	Costs OR Cost Analysis OR Affordabilities OR Affordability OR Cost OR Cost Analyses OR Cost Analysis OR Cost Comparison OR Cost Comparisons OR Cost	Custos OR Análise de Custo OR Acessibilidade Financeira OR Análise de Custo em Saúde OR Análise de Custos OR Análise de Minimização de Custo OR Análise de	Costos OR Análisis de Costo OR Análisis de Costo-Minimización OR Análisis de Minimización de Costo OR Análisis de Minimización de Costos OR

	Measure OR Cost Measures OR Cost Minimization Analysis OR Cost-Minimization Analyses OR Cost-Minimization Analysis OR Costs OR Costs and Cost Analyses OR Pricing	Minimização de Custos OR Capacidade Aquisitiva OR Capacidade de Pagar pelos Serviços de Saúde OR Capacidades de Pagar pelos Serviços de Saúde OR Comparação de OR Custos OR Custo OR Custos e Análises de Custo OR Medidas de Custo OR Precificação	Asequibilidad OR Asequibilidades

Findings In 21/07/2022.

**Language of descriptors**	**PubMed**	**Embase**	**LILACS**	**SciELO**
**English**	**3257**	**3615**	**207**	**56**
**Portuguese**	**18**	**27**	**159**	**13**
**Spanich**	**25**	**15**	**177**	**32**

TOTAL: 7601

DUPLICATED: 2297

TOTAL AFTER REMOVING DUPLICATES in mendeley:5380

Duplicate finds in zotero:145

TOTAL AFTER REMOVING DUPLICATES in zotero: 5235

Duplicate finds in rayyan: 16

TOTAL FOR READING TITLES AND ABSTRACTS:5137

Query: (((Chickungunya) OR (Chickungunya Fever) OR (Chickungunya Fevers) OR (Chikungunya Fevers) OR (Chikungunya Virus Infection) OR (Chikungunya Virus Infections)) AND ((Cross-Sectional Studies) OR (Cross Sectional Analyses) OR (Cross Sectional Analysis) OR (Cross Sectional Studies) OR (Cross Sectional Survey) OR (Cross-Sectional Analyses) OR (Cross-Sectional Analysis) OR (Cross-Sectional Study) OR (Cross-Sectional Survey) OR (Cross-Sectional Surveys) OR (Disease Frequency Survey) OR (Disease Frequency Surveys) OR (Prevalence Studies) OR (Prevalence Study) OR (Cohort Studies) OR (Cohort Analyses) OR (Cohort Analysis) OR (Historical Cohort Studies) OR (Historical Cohort Study) OR (Incidence

Studies) OR (Incidence Study) OR (Morbidity) OR (Morbidities) OR (Mortality) OR (Case) OR (Fatality Rate) OR (Case Fatality Rates) OR (Crude Death Rate) OR (Crude Death Rates) OR (Crude) OR (Mortality Rate) OR (Crude Mortality Rates) OR (Death Rate) OR (Death Rates) OR (Mortalities) OR (Mortality Rate) OR (Mortality Rates) OR (Disability-Adjusted Life Years) OR (DALY) OR (DALYs) OR (Disability Adjusted Life Years) OR (Disability-Adjusted Life Year) OR (Years Lived With Disability) OR (Seroepidemiologic Studies) OR (Seroepidemiologic Study) OR (Seroepidemiological Studies) OR (Seroepidemiological Study) OR (Seroprevalence) OR (Seroprevalences) OR (Cost of Illness) OR (Burden Of Disease) OR (Burden Of Diseases) OR (Burden of Illness) OR (Cost of Disease) OR (Cost of Sickness) OR (Disease Burden) OR (Disease Burdens) OR (Disease Cost) OR (Disease Costs) OR (Economic Burden of Disease) OR (Illness Burden) OR (Illness Burdens) OR (Illness Cost) OR (Illness Costs) OR (Sickness Cost) OR (Sickness Costs) OR (Cost Allocation) OR (Cost Allocations) OR (Cost Apportionment) OR (Cost Apportionments) OR (Cost Shifting) OR (Cost Shiftings) OR (Health Care Costs) OR (Health Care Cost) OR (Health Cost) OR (Health Costs) OR (Healthcare Cost) OR (Healthcare Costs) OR (Medical Care Cost) OR (Medical Care Costs) OR (Treatment Cost) OR (Treatment Costs) OR (Drug Costs) OR (Drug Cost) OR (Direct Service Costs) OR (Direct Service Cost) OR (Hospital Costs) OR (Hospital Cost) OR (Cost Efficiency Analysis) OR (Cost-Benefit Analysis) OR (Benefits and Costs) OR (Cost and Benefit) OR (Cost Benefit) OR (Cost Benefit Analyses) OR (Cost Benefit Analysis) OR (Cost Benefit Data) OR (Cost Effectiveness) OR (Cost Effectiveness Analysis) OR (Cost Utility Analysis) OR (Cost-Benefit Analyses) OR (Cost-Benefit Data) OR (Cost-Effectiveness Analysis) OR (Cost-Utility Analyses) OR (Cost-Utility Analysis) OR (Costs and Benefits) OR (Economic Evaluation) OR (Economic Evaluations) OR (Marginal Analyses) OR (Marginal Analysis) OR (Cost-Effectiveness Evaluation) OR (Costs and Cost Analysis) OR (Affordabilities) OR (Affordability) OR (Cost) OR (Cost Analyses) OR (Cost Analysis) OR (Cost Comparison) OR (Cost Comparisons) OR (Cost Measure) OR (Cost Measures) OR (Cost Minimization Analysis) OR (Cost-Minimization Analyses) OR (Cost-Minimization Analysis) OR (Costs) OR (Costs and Cost Analyses) OR (Pricing)))

(((Febre de Chikungunya) OR (Chicungunha) OR (Chikungunya) OR (Febre Chickungunya) OR (Febre Chikungunya) OR (Febre do Chikungunya) OR (Infecção pelo Vírus Chikungunya) OR (Infecção por Vírus Chikungunya)) AND ((Estudos Transversais) OR (Estudos de Corte Transversal) OR (Estudos de Prevalência) OR (Estudos Seccionais) OR (Levantamentos de Frequência de Doenças OR Estudos de Coortes) OR (Análise de Coortes) OR (Estudos de Incidência) OR (Estudos Históricos de Coortes) OR (Morbidade) OR (Frequência) OR (Morbidez) OR (Morbilidade) OR (Taxa de Morbidade) OR (Mortalidade) OR (Coeficiente de Mortalidade) OR (Estatísticas de Mortalidade) OR (Índice de Casos Fatais) OR (Índice de Fatalidade) OR (Índice de Letalidade) OR (Índice de Mortalidade) OR (Letalidade) OR (Taxa de Casos Fatais) OR (Taxa de Fatalidade) OR (Taxa de Letalidade) OR (Taxa de Mortalidade) OR (Anos de Vida Ajustados pela Incapacidade) OR (Anos de Vida Perdidos por Incapacidade) OR (Anos Vividos com Deficiência) OR (DALYs) OR (Estudos Soroepidemiológicos) OR (Soroepidemiologia) OR (Soroprevalência) OR (Efeitos Psicossociais da Doença) OR (Carga da Doença) OR (Carga das Doenças) OR (Carga de Doença) OR (Custo da Doença) OR (Custo da Doença para o Paciente) OR (Custos da Doença) OR (Fardo da Doença) OR (Fardos Relativos à Doença) OR (Ônus da

Doença) OR (Peso da Doença) OR (Alocação de Custos) OR (Distribuição de Custos) OR (Substituição de Custos) OR (Custos de Cuidados de Saúde) OR (Custos de Cuidados Médicos) OR (Custos de Tratamento) OR (Custos de Medicamentos) OR (Despesa com Fármacos) OR (Despesa com Medicamento) OR (Despesa com Medicamentos) OR (Despesas com Fármacos) OR (Despesas com Medicamentos) OR (Gasto com Fármacos) OR (Gasto com Medicamentos) OR (Custos Diretos de Serviços) OR (Custo Direto) OR (Custos Hospitalares) OR (Análise Custo-Eficiência) OR (Análise Custo-Benefício) OR (Análise de Custo-Benefício) OR (Análise de Custo-Efetividade) OR (Análise de Custo-Utilidade) OR (Análise Marginal) OR (Avaliação Econômica) OR (Custo-Benefício) OR (Custo-Efetividade) OR (Dados de Custo-Benefício) OR (Avaliação de Custo-Efetividade) OR (Custos) OR (Análise de Custo) OR (Acessibilidade Financeira) OR (Análise de Custo em Saúde) OR (Análise de Custos) OR (Análise de Minimização de Custo) OR (Análise de Minimização de Custos) OR (Capacidade Aquisitiva) OR (Capacidade de Pagar pelos Serviços de Saúde) OR (Capacidades de Pagar pelos Serviços de Saúde) OR (Comparação de) OR (Custos) OR (Custo) OR (Custos e Análises de Custo) OR (Medidas de Custo) OR (Precificação)))

(((Fiebre Chikungunya) OR (Chickungunya) OR (Chikungunya) OR (Chikunguña) OR (Fiebre Chickungunya) OR (Fiebre Chikunguña) OR (Fiebre de Chikungunya) OR (Fiebre de Chikunguña) OR (Fiebre por el Virus del Chikungunya) OR (Infección por el Virus Chikungunya) OR (Infección por Virus Chikungunya)) AND ((Estudios Transversales) OR (Encuestas de Frencuencia de Enfermedades) OR (Estudios de Corte Transversal) OR (Estudios de Prevalencia) OR (Estudios Seccionales) OR (Estudios de Cohortes) OR (Análisis de Cohortes) OR (Estudios de Incidencia) OR (Estudios Históricos de Cohortes) OR (Morbidad) OR (Mortalidad) OR (Estadísticas de Mortalidad) OR (Índice de Casos Fatales) OR (Índice de Fatalidad) OR (Índice de Letalidad) OR (Índice de Mortalidad) OR (Letalidad) OR (Tasa de Casos Fatales) OR (Tasa de Fatalidad) OR (Tasa de Letalidad) OR (Tasa de Mortalidad) OR (Años de Vida Ajustados por Discapacidad) OR (Años de vida Ajustados por la Incapacidad) OR (Años de Vida Perdidos por Incapacidad) OR (Años Vividos con Discapacidad) OR (DALYs) OR (Estudios Seroepidemiológicos) OR (Seroepidemiología) OR (Seroprevalencia) OR (Costo de Enfermedad) OR (carga de la enfermedad) OR (carga de las dolencias) OR (carga de las enfermedades) OR (carga de morbimortalidad) OR (coste de la enfermedad para el paciente) OR (costes de las dolencias) OR (Asignación de Costos) OR (desviación de costes) OR (distribución de costes) OR (imputación de costes) OR (costes de la atención a la salud) OR (costes de la asistencia sanitaria) OR (costes de la atención médica) OR (costes del tratamiento) OR (Costos de los Medicamentos) OR (coste de farmacia) OR (Costos Directos de Servicios) OR (Coste directo de los servicios) OR (Costos de Hospital) OR (costes hospitalarios) OR (Análisis Costo-Eficiencia) OR (Análisis Costo-Beneficio) OR (análisis de coste-utilidad) OR (análisis de costo-utilidad) OR (Coste-beneficio) OR (Coste-efectividad) OR (datos de coste-beneficio) OR (evaluación económica) OR (relación coste-eficacia) OR (Costos) OR (Análisis de Costo) OR (Análisis de Costo-Minimización) OR (Análisis de Minimización de Costo) OR (Análisis de Minimización de Costos) OR (Asequibilidad) OR (Asequibilidades)))

2. Searches/Bases: SciELO, Medline/Pubmed, Embase and LILACS

- The Period: last 15 years (2007 - 2022)
- Languages: Portuguese and English and Spanish
- Add articles from the included references.

3. Selection of studies (Mendeley for organization and Rayyan for selection):

- The eligibility criteria
- Exclusion criteria
- duplication

NOTE: inclusion in PROSPERO (PRISMA-P guide https://www.prisma-statement.org//documents/PRISMA-P-checklist.pdf)

4. Data extraction (selection and programming):
The Build an Excel table (guide How to prepare a systematic review of economic evaluations for informing evidence-based healthcare decisions: data extraction, risk of bias, and transferability https://www.tandfonline.com/doi/pdf/10.1080/14737167.2016.1246961?needAccess=true)

5. Assessment of the methodological quality of the included studies (Risk of bias):
- The Analysis of risk of bias epidemiological studies: Newcastle-Ottawa scale https://www.ohri.ca/programs/clinical_epidemiology/nosgen.pdf
- Economic studies (guide options): Script for reporting economic evaluation studies http://scielo.iec.gov.br/scielo.php?script=sci_arttext&pid=S1679-49742017000400895
- Systematic reviews and meta-analyses: AMSTAR 2

6. Strategy for data synthesis: qualitative discussion, without meta-analysis

7. Writing:
- The Writing of the article (following the PRISMA checklist https://prisma-statement.org/documents/PRISMA%202020%20checklist%20EUROPEAN%20PORTUGUESE.pdf)
- Abstract writing (following the PRISMA checklist https://prisma-statement.org//documents/PRISMA%202020%20abstract%20checklist%20EUROPEAN%20PORTUGUESE.pdf)
- Make a flowchart (following the PRIMA guide https://prisma-statement.org/documents/PRISMA%202020%20flow%20diagram%20EUROPEAN%20PORTUGUESE.pdf). Template: https://prisma-statement.org/documents/PRISMA_2020_flow_diagram_new_SRs_v1.docx
