# Supplementary material for: Epidemiology and Economic Burden of Chikungunya: A Systematic Literature Review
Source: Trop Med Infect Dis. 2023 May 31;8(6):301. doi: 10.3390/tropicalmed8060301 (PMC10302198; doi:10.3390/tropicalmed8060301)
Supplement: Supplementary file 1 [file tropicalmed-08-00301-s001.zip › SM_3_QUALITY_ASSESSMENT_ROB.pdf]

Tables S3: Quality assessment of included studies

NEWCASTLE – OTTAWA: Escala de Acesso de Qualidade										
	Doran et al (2022)	Gerarsin P et al (2011)	Bertolotti et al A (2020)	Chopra A (2012)	Gordon A et al (2018)	Moyen N et al (2011)	Soumahoro Mk et al (2009)	Gerardin P et al (2011)	Bertolotti A et al (2020)	Couzigou B et al (2018)
Representativeness of the Exposed Cohort	No	Yes	YES	yes	yes	yes	yes	yes	yes	yes
Selection of the Non-Exposed Cohort	No	yes	yes	yes	yes	yes	yes	yes	yes	yes
Ascertainment of Exposure	Yes	yes	yes	yes	yes	yes	yes	yes	yes	yes
Demonstration That Outcome of Interest Was Not Present at Start of Study	Yes	yes	yes	no	no	no	yes	yes	yes	yes
Comparability of Cohorts on the Basis of the Design or Analysis	Yes	No	no	yes	no	no	yes	yes	yes	yes
Assessment of Outcome	Yes	yes	yes	yes	yes	yes	yes	yes	yes	yes
Was Follow-Up Long Enough for Outcomes to Occur	Yes	yes	yes	yes	yes	yes	yes	yes	yes	yes
Adequacy of Follow Up of Cohorts	NO	yes	yes	no	yes	no	yes	yes	yes	yes

The A Measurement Tool to Assess Systematic Reviews (AMSTAR)						
	Simo FBN et al. (2019)	Rodríguez-Morales AJ. 2016	Idris Nasir Abdullahi, (2020)	Badawi A. 2018	Li Z et al (2021)	Thompson R. (2020)
	High	High	Critically low	Moderate	Moderate	Low

The Joanna Briggs Institute (JBI)									
	Were the criteria for inclusion in the sample clearly defined?	Were the study subjects and the setting described in detail?	Was the exposure measured in a valid and reliable way?	Were objective, standard criteria used for measurement of the condition?	Were confounding factors identified?	Were strategies to deal with confounding factors stated?	Were the outcomes measured in a valid and reliable way?	Was appropriate statistical analysis used?	
Murhekar M, et al. (2019)	yes	yes	yes	yes	Not applicable	Not applicable	yes	yes	6/8
Mercado-Reyes M, et al. (2019)	yes	yes	yes	yes	Not applicable	Not applicable	yes	yes	6/8
Vidal ERN, et al. (2022)	yes	yes	yes	yes	yes	yes	yes	yes	8/8

Ramachandran V, et al (2012)	yes	yes	yes	yes	yes	yes	yes	yes	8/8
Kinimi E, et al. (2018)	yes	yes	yes	yes	Not applicable	Not applicable	yes	yes	6/8
Luvai EAC et al. (2021)	yes	yes	yes	yes	yes	yes	yes	yes	8/8
Joshi P, et al. (2020)	yes	yes	yes	yes	Not applicable	Not applicable	yes	yes	6/8
Mathew AJ, et al. (2011)	yes	yes	yes	yes	yes	yes	yes	yes	8/8
Mwanyika GO, et al. (2021)	yes	yes	yes	yes	yes	yes	yes	yes	8/8
Mease LE, et al. (2011)	yes	yes	yes	yes	yes	yes	yes	yes	8/8
Khongwichit S, et al. (2021)	yes	yes	yes	yes	Not applicable	Not applicable	yes	yes	6/8
Simião AR et al. (2019)	yes	yes	yes	yes	Not applicable	Not applicable	yes	yes	6/8
Renault P, et al. (2007)	yes	yes	yes	yes	Not applicable	Not applicable	yes	yes	6/8

Khatun S, et al (2015)	yes	yes	yes	yes	Not applicable	Not applicable	yes	yes	6/8
Nicacio JM, et al. (2021)	yes	yes	yes	yes	Not applicable	Not applicable	yes	yes	6/8
Hertz JT, et al. (2012)	yes	yes	yes	yes	Not applicable	Not applicable	yes	yes	6/8
Ray P, et al. (2012)	yes	yes	yes	yes	Not applicable	Not applicable	yes	yes	6/8
Hossain MS, et al. (2018)	yes	yes	yes	yes	yes	yes	yes	yes	8/8
Huits R, et al. (2018)	yes	yes	yes	yes	yes	yes	yes	yes	8/8
Henry M. (2017)	yes	yes	yes	yes	Not applicable	Not applicable	yes	yes	6/8
Barr KL, et al. (2018)	yes	yes	yes	yes	unclear	unclear	yes	no	5/8
Aubry M et al. (2020)	no	no	yes	yes	no	no	yes	yes	4/8
Dorléans F, et al. (2018)	yes	yes	yes	yes	no	no	yes	yes	6/8
Barreto, MCA et al.	no	yes	yes	yes	Not	Not	yes	yes	5/8

(2021)					applicable	applicable			
Ekong PS, et al. (2022)	yes	no	yes	yes	no	yes	yes	yes	6/8
Chis Ster I, et al. (2020)	yes	yes	yes	yes	yes	yes	yes	yes	8/8
Teixeira MG, et al. (2021)	yes	yes	yes	yes	yes	yes	yes	yes	8/8
Economopoulou A, et al. (2009)	yes	yes	yes	yes	yes	yes	yes	yes	8/8
Vongpunsawad S, et al. (2017)	yes	yes	yes	yes	no	no	yes	yes	6/8
Endale A, et al. (2020)	yes	yes	yes	yes	yes	yes	yes	yes	8/8
Badar N, et al. (2021)	yes	no	yes	yes	no	no	yes	Not applicable	4/5
Hennessey MJ, et al. (2018)	yes	yes	yes	yes	yes	yes	yes	yes	8/8
Kumar, MS et al. (2021)	yes	yes	yes	yes	yes	yes	yes	yes	8/8
Azami NA, et al (2013)	yes	no	yes	yes	yes	yes	yes	yes	7/8
Chattopadhyay, S.	yes	no	yes	yes	no	no	yes	Not	4/8

(2016)								applicable	
de Andrade DC (2010)	yes	yes	yes	yes	yes	yes	yes	yes	8/8
Sissoko D (2008)	yes	yes	yes	yes	yes	yes	yes	yes	8/8
Sergon K. (2008)	yes	yes	yes	yes	no	unclear	yes	unclear	5/8
Frutuoso LCV. (2020)	yes	yes	yes	yes	no	unclear	yes	unclear	5/8
Gallian P. (2017)	yes	yes	yes	yes	yes	yes	yes	yes	8/8
Perissé ARS (2020)	yes	yes	yes	yes	yes	yes	yes	yes	8/8
Antonio VS (2018) eu queria	no	yes	yes	yes	no	no	yes	yes	5/8
Dias JP (2018)	yes	yes	yes	yes	no	no	yes	yes	6/8
Cunha RV (2017)	yes	yes	yes	yes	no	no	yes	yes	6/8
Barreto FKA (2020)	yes	yes	yes	yes	no	no	yes	yes	6/8
Crosby L (2016)	unclear	no	unclear	yes	no	no	yes	unclear	2/8
Murhekar M (2019)	unclear	no	unclear	yes	Not applicable	Not applicable	yes	unclear	2/8

Roteiro CHEERS											
	Vázquez-Cruz I. (2018)	Teich V. (2017)	Krishnamoorthy K (2009)	Cardona-Ospina, J. A (2015)	Feldstein LR. (2019)	Alvis-Zakzuk NJ. (2018)	Mora-Salamanca AF. (2020)	Soumahoro M (2011)	Schilte C (2013)	Gopalan SS (2009)	Cordona-Ospina J et al 2015
Introduction											
Background and objectives	yes	yes	yes	yes	yes	yes	yes	yes	yes	yes	yes
Methods											
Target-population and subgroups	yes	no	yes	yes	yes	yes	yes	no	yes	yes	yes
Context and location	yes	yes	yes	yes	no	no	yes	yes	yes	yes	yes
Study perspective	yes	yes	yes	yes	yes	yes	yes	yes	yes	yes	yes
Comparators	no	yes	no	yes	no	no	yes	no	yes	no	yes
Time horizon	yes	yes	yes	no	yes	no	yes	yes	yes	yes	yes
Discount rate	no	no	no	no	yes	yes	no	no	no	no	yes
Choice of the health	yes	yes	yes	yes	yes	yes	yes	yes	yes	yes	yes

outcome											
Measurement of effectiveness	no	no	no	no	no	no	no	no	no	no	no
	no	yes	yes	no	no	no	no	no	no	no	no
Measurement and valuation of results based on preferences	no	yes	yes	no	no	no	no	yes	no	no	no
Estimating resources and costs	no	yes	yes	yes	yes	yes	yes	yes	no	no	yes
	yes	yes	yes	yes	no	yes	yes	yes	no	yes	yes
Currency, price date and conversion	yes	yes	yes	yes	yes	yes	yes	yes	no	yes	yes
Choice of model	yes	yes	yes	no	no	no	yes	yes	yes	yes	yes
Assumptions	yes	yes	yes	no	no	no	yes	no	yes	yes	yes
Analytical methods	yes	yes	yes	no	no	yes	yes	yes	yes	yes	yes
Results											
Study parameters	yes	yes	yes	yes	yes	yes	yes	yes	yes	yes	yes
Incremental costs	yes	yes	yes	yes	no	no	yes	yes	yes	yes	yes

and outcomes											
Characterizing uncertainty	yes	yes	no	no	no	no	no	no	no	no	no
	yes	yes	no	no	no	no	no	no	no	no	no
Characterizing heterogeneity	yes	yes	no	no	no	no	yes	no	no	no	no
Discussion											
Study findings, limitations, generalizability, and current knowledge	yes	yes	yes	yes	yes	no	yes	yes	yes	yes	yes
Sources of funding	no	no	no	no	yes	yes	yes	yes	yes	no	yes
Conflicts of interest	yes	no	no	yes	yes	yes	yes	yes	yes	no	yes
